# Integrated analysis of organelle RNA editing and DYW- type PPR genes identifies a candidate regulator of plastid *ndhD*-878 editing under drought stress in soybean

**DOI:** 10.3389/fpls.2026.1879625

**Published:** 2026-07-06

**Authors:** Yangyang Liu, Bing Wang, Zhaoming Qi, Qingshan Chen, Yi Liu, Shaodong Wang, Sui Wang

**Affiliations:** 1 National Key Laboratory of Smart Farm Technologies and Systems, Northeast Agricultural University, Harbin, China; 2 Key Laboratory of Soybean Biology of Chinese Education Ministry, Northeast Agricultural University, Harbin, China; 3 College of Landscape and Tourism, Hebei Agricultural University, Baoding, China; 4 State Key Laboratory of Tree Genetics and Breeding, Northeast Forestry University, Harbin, China; 5 Innovative Center of Molecular Genetics and Evolution, Guangzhou University, Guangzhou, China

**Keywords:** drought stress, organelle, PPR gene family, RNA editing, soybean

## Abstract

RNA editing in plant organelles is an important post-transcriptional process that helps maintain organelle gene function and may contribute to plant responses to environmental stress. However, drought-responsive RNA editing events and their regulatory factors remain poorly understood in soybean. Pentatricopeptide repeat (PPR) proteins, especially DYW-type PPR proteins, are key components of plant organellar RNA editing complexes and often determine the recognition of specific editing sites. In this study, we analyzed plastid and mitochondrial RNA editing in the soybean cultivar Dongfudou 3 under natural dehydration stress and investigated DYW-type PPR genes as potential regulators. A total of 60 plastid and 684 mitochondrial RNA editing sites were identified, most of which were located in coding regions and caused nonsynonymous C-to-U changes. Under drought stress, 19 plastid and 71 mitochondrial coding-region sites showed marked changes in editing efficiency, suggesting dynamic regulation of organellar RNA editing by water deficit. Genome-wide analysis identified 822 high-confidence soybean PPR genes, and DYW-type members were further screened using drought-responsive transcriptome data and predicted editing targets. Gm_DFD3_00451 was selected as a candidate regulator because it showed drought-responsive expression, was associated with the plastid *ndhD*-878 editing site and was confirmed to localize in plastids. The RNA EMSA result supports the interaction between Gm_DFD3_00451 and the *ndhD*-878 upstream RNA probe. In CRISPR-edited soybean hairy roots, disruption of *Gm_DFD3_00451* reduced *ndhD*-878 editing efficiency and altered drought-related physiological traits. These results suggest that Gm_DFD3_00451 participates in plastid RNA editing during drought stress in soybean.

## 1 Introduction

Soybean (*Glycine max* (L.) Merr.), domesticated from wild soybean in East Asia millennia ago, is an important crop for plant protein and edible oil production (Kudełka W et al., 2021). Drought stress is one of the major environmental factors that limits soybean growth, yield, and seed quality. At the physiological level, water deficit reduces water uptake, disturbs osmotic balance, limits photosynthesis and growth, and promotes oxidative damage. These changes are accompanied by broad molecular adjustments that help plants maintain cellular homeostasis and adapt to water-deficit conditions (Wang C et al., 2025). Therefore, understanding drought-responsive molecular regulation in soybeans is important for improving stress adaptation and breeding drought-tolerant cultivars.

At the molecular level, drought responses are controlled by multiple regulatory layers, including transcriptional, post-transcriptional, translational, and metabolic regulation. Among these processes, RNA editing is an important post-transcriptional modification in plant organelles (Saeedi et al., 2026; Shi et al., 2024). In higher plants, RNA editing mainly occurs in plastid and mitochondrial transcripts and usually converts cytidine to uridine (Small ID et al., 2020). Many editing events are located in coding regions and can change codons, restore conserved amino acids, or maintain the function of organelle-encoded proteins (Hao W et al., 2021). Thus, organellar RNA editing is closely linked to plastid and mitochondrial gene expression, organelle development, respiration, photosynthesis, and stress-related metabolism. Compared with plastid genomes, plant mitochondrial genomes are often larger and structurally more complex, including multipartite or multi-chromosomal organizations in some species, which highlights the importance of accurate organellar reference genomes for mitochondrial transcriptome and RNA editing analyses (Bi et al., 2022). Extensive research indicates that RNA editing is not only required for normal organelle function but may also respond to environmental signals.

Accumulating evidence shows that organellar RNA editing is sensitive to abiotic stress and that different stresses can alter editing efficiency in a site-specific manner. In soybean, salt stress was shown to change chloroplast RNA editing levels in transcripts such as *ndhA*, *ndhB*, *rps14*, and *rps16*, suggesting that stress-regulated editing may help maintain chloroplast electron transport and translation-related functions under saline conditions (Rodrigues NF et al., 2017). In *Arabidopsis*, loss of RARE1-mediated editing of the chloroplast *accD* transcript impaired fatty acid biosynthesis and reduced heat tolerance, indicating that a single editing event can affect membrane stability under stress (Huang C et al., 2023). More directly related to drought, genome-wide analysis in rice showed that mitochondrial RNA editing responses to drought are site-specific and genotype-specific, and that two mitochondrial PPR proteins, PPR035 and PPR406, regulate specific editing sites associated with drought-related traits (Luo Z et al., 2022). These studies suggest that stress-responsive RNA editing may act through at least two connected mechanisms: by changing the coding potential and function of organelle-encoded proteins, and by modulating organelle processes such as photosynthetic electron transport, respiration, membrane homeostasis, and reactive oxygen species balance. However, how drought affects plastid and mitochondrial RNA editing in soybean, and which factors regulate key drought-responsive editing sites, remain unclear. The search for plant RNA editing regulatory factors has largely focused on the protein complexes that mediate organellar RNA editing. Pentatricopeptide repeat (PPR) proteins are key components of these complexes and usually provide target-site recognition through tandem PPR motifs (Cheng S et al., 2016). In many cases, PLS-type and DYW-type PPR proteins recognize sequences upstream of the edited cytidine, while other editing factors, such as MORF/RIP (Xiong Y et al., 2022), ORRM (Shi X et al., 2017), OZ1 (Gipson AB et al., 2020), and PPO1 proteins (Zhang F et al., 2014), support editing complex assembly or activity. The DYW domain is closely related to C-to-U editing activity, making DYW-type PPR proteins strong candidates for identifying regulators of specific organellar editing sites (Okuda K et al., 2009). Recent structural and biochemical studies further support the model that PPR domains bind target RNA sequences and position the DYW domain for cytidine deamination (Takenaka M et al., 2021).

In this study, we used the soybean cultivar Dongfudou 3 (*Glycine max*) to investigate drought-responsive organellar RNA editing. We first analyzed plastid and mitochondrial RNA editing under drought stress and focused on editing sites with significantly altered editing efficiency. We then performed genome-wide identification and characterization of soybean PPR genes, with particular attention to DYW-type members, and integrated drought-responsive transcriptome data with target prediction to screen candidate editing regulators. One candidate PPR gene and its predicted target editing site were further selected for validation. Subcellular localization and RNA EMSA were used to examine its plastid targeting and RNA-binding ability, whereas CRISPR-edited soybean hairy roots were used as a rapid system for preliminary functional analysis. Through this design, we aimed to determine how drought stress affects organellar RNA editing in soybean and to provide preliminary evidence for the role of a key PPR protein in regulating RNA editing at its target site.

## 2 Materials and methods

### 2.1 Plant materials and growth conditions

The beany flavor-free soybean cultivar Dongfudou 3 was used in this study. This cultivar was developed in our laboratory. Its nuclear and organellar genomes have been sequenced and assembled (unpublished; plastid genome GenBank accession No. OR664111.1; mitochondrial genome GenBank accession No. OR687435.1). For RNA editing site analysis, plants were grown in soil. Seeds were surface-sterilized, washed, and soaked in water for 6–24 h to promote imbibition. Once swollen, the seeds were evenly spread on perforated trays and kept moist for germination. When the sprouts reached 5–6 cm in height, they were transplanted into a soil mixture composed of soil, vermiculite, and perlite (5:2:1, v/v/v). Transgenic hairy roots were maintained hydroponically (Huang et al., 2022; Kereszt A et al., 2007).

### 2.2 Drought stress treatment

Drought stress was imposed at the V3 growth stage. For RNA editing analysis, soil water status was estimated using the pot-weighing method and expressed as a percentage of field capacity. Control plants were maintained at approximately 80–85% field capacity. For drought treatment, irrigation was withheld until soil water content decreased to 50–55% field capacity on day 5, when plants showed mild drought symptoms (Wang C et al., 2025).

Fully expanded young leaves were then harvested, immediately frozen in liquid nitrogen, and stored at -80°C. For functional validation, transgenic hydroponic hairy roots of the empty-vector control (EV) and *Gm_DFD3_00451*-edited line KO-4 were treated with 0% or 5% PEG 6000 (Qi et al., 2023). Each treatment included three biological replicates.

### 2.3 Transcriptome sequencing

Samples were transported to library construction and sequencing. Total RNA was extracted from leaf tissue using the CTAB method. Strand-specific lncRNA libraries were prepared and sequenced on the Illumina NovaSeq X10 platform by Wuhan Farsight Gene Information Co., Ltd. Raw reads were assessed with FastQC (v0.11.9) and subsequently filtered using fastp (v0.23.2) with the parameters -f 8, -t 2, -n 0, -l 140, -q 20, and -u 20. The filtered data were re-evaluated with FastQC to confirm data quality before downstream analyses (Chen S et al., 2018).

### 2.4 Statistical analysis of RNA editing sites under drought stress treatments

To identify RNA editing sites in the plastid and mitochondrial genomes of Dongfudou 3 under drought stress, clean RNA-seq reads from each sample were aligned to the corresponding organellar reference genomes using HISAT2 (v2.1.0) (Kim D et al., 2019). The alignments were sorted and indexed with SAMtools (v1.9), and candidate variants were called using the BCFtools (v1.12) mpileup and call commands (Li H et al., 2009; Danecek P et al., 2021). Read depth and editing efficiency were calculated for each candidate site using custom scripts, and editing efficiency was defined as the proportion of reads supporting the edited base among all reads covering that site. To reduce false positives caused by sequencing errors, genomic polymorphisms, and nuclear organellar fragments, genomic DNA reads of Dongfudou 3 were aligned to the nuclear, plastid, and mitochondrial reference genomes, and candidate sites supported by gDNA reads or showing ambiguous mapping were excluded, following the strategy used in our previous RNA editing analysis with minor modifications (Wang et al., 2018). Candidate sites were also manually inspected using SAMtools tview. Only C-to-U editing sites with an editing efficiency greater than 20% and reliable read support were retained for subsequent analysis. The retained sites were annotated using VCFtools (v4.0) and SnpEff (v5.4) to determine their genomic locations, functional effects, and predicted amino acid changes (Danecek P et al., 2011; Cingolani P et al., 2012).

Editing efficiency values were calculated from three biological replicates. Differences in editing efficiency between the control and drought treatment were evaluated using the Kruskal-Wallis test, and coding-region sites with both statistical significance and an editing efficiency difference of at least 30 percentage points were considered drought-responsive editing sites.

### 2.5 PPR protein site prediction and candidate site identification

Transcriptome data were normalized with edgeR, and differentially expressed genes (DEGs) were identified with thresholds of |log_2_FC| ≥ 1 and FDR< 0.05. From these DEGs, representative DYW-type PPR genes were selected for validation by quantitative real-time PCR (qRT−PCR). Leaf cDNA from drought-stressed and control plants was used as the template, and amplification was performed with SYBR Green. The relative expression levels were calculated using the 2^-ΔΔCt^ method, and all primer sequences are provided in **Supplementary Table 1**.

Protein sequences were analyzed with the PPRCODE database (Yan J et al., 2019) and Prosite Psscan (De Castro E et al., 2006) to detect PPR motifs. Based on the established principle that the amino acids at positions 5 and 35 of each repeat dictate nucleotide specificity, only proteins containing at least eight tandem PPR repeats were considered competent for RNA binding (Barkan A et al., 2012; Yagi Y et al., 2013). Starting from the C terminus, each repeat was aligned in reverse to the nucleotide immediately upstream of its target editing site, and these upstream nucleotides were concatenated to generate candidate recognition sequences (Kobayashi T et al., 2019).

Plastid and mitochondrial genome sequences were indexed in BioEdit and queried using BLASTN with an E value threshold of 0.05 and m8 output format. Predicted targets from PLS subfamily members, particularly DYW, E, and E+ types, were collected, redundancy was removed, and the resulting set was intersected with experimentally identified editing sites under stress.

### 2.6 RNA EMSA verification of Gm_DFD3_00451 binding to the ndhD-878 upstream sequence

The coding sequence of *Gm_DFD3_00451* was cloned into the pET28a vector using *EcoR*I and *Hind*III and transformed into *E. coli* BL21(DE3). Protein expression was induced with 0.2 mM IPTG at 16°C for 16 h, and the recombinant protein was purified using the BeaverBeads^®^ IDA-Nickel Kit (Cat. No. 70501-K10). The binding reaction was performed using the [BersinBio™ RNA-EMSA Kit (BersinBio)] (Cat. No. Bes5107) with the RNA probe (**Supplementary Table 1**). Binding of RNA Probe and Gm_DFD3_00451 proteins was detected using the BersinBio™ RNA-EMSA Kit (BersinBio) according to the operating manual. For each reaction, 12.5 nM biotin-labeled RNAs were incubated with 200 ng purified protein. The controls included a free probe control (to confirm the position of unbound RNA) and cold probe competition control (20×, 50×, and 100× excess unbiotinylated probe, to preliminarily assess binding specificity).

### 2.7 CRISPR knockout vector construction and hairy root transformation

Target-specific single guide RNA (sgRNA) sequences were designed using the CRISPR-P online platform (http://crispr.hzau.edu.cn/cgi-bin/CRISPR2/CRISPR), with two sgRNAs designed for the target gene. Pairs of oligonucleotides with 5′ adapters (TGCA for the sense strand and AAAC for the antisense strand) compatible with the *Bsa*I sites of the pGES401 vector were synthesized, annealed into double-stranded DNA, and ligated into *Bsa*I-linearized pGES401 at a molar ratio of 1:5. After verification by sequencing, the resulting recombinant vector was introduced into *Agrobacterium rhizogenes* K599.

Hairy root transformation was performed following a modified protocol based on Kereszt et al. (Kereszt A et al., 2007) and Jing F et al (Jing F et al., 2025). Soybean seeds were surface sterilized with 75% ethanol for 5 min, treated with 15% H_2_O_2_ for 12 min at 28°C with shaking at 110 rpm, rinsed thoroughly, and soaked in ultrapure water for 16 h to promote radicle emergence. *Agrobacterium rhizogenes* K599 harboring the target vector was grown to log phase (OD^600^ = 1.0), harvested, and resuspended in LCCM induction medium (pH 5.4) containing 40 mg·L^-1^ acetosyringone. Pre-treated seeds were dissected to obtain hypocotyls and cotyledons, which were immersed in the bacterial suspension for 20 min, blotted dry, and co-cultured in the dark at 25 ± 1°C for 48 h on medium supplemented with cysteine, DTT, and acetosyringone. Explants were then transferred to hairy root induction medium containing ticarcillin and cefotaxime (pH 5.7) and cultured under a 16 h light/8 h dark photoperiod (100-150 μmol·m^-2^·s^-1^, 23-25°C) for 7 days. When the emerged roots reached 5–10 cm in length, they were carefully transferred to hydroponic culture for 3 days before being planted in soil.

For knockout validation, genomic DNA was extracted from 0.2 g of hairy root tissue. PCR amplification was performed using primers 00451-Cas-F and 00451-Cas-R (**Supplementary Table 1**), and the amplicons were cloned into a T vector (pMD19-T) for sequencing to assess both editing efficiency and the nature of the induced mutations.

### 2.8 Site editing efficiency detection

Primers were designed to amplify a region within 300 bp upstream and downstream of the target RNA editing site. PCR was performed using cDNA from knockout positive hairy roots. The PCR fragments were then ligated into a T−vector (pMD19-T) and transformed into E. *coli* DH5α competent cells. Thirty single colonies were randomly picked and analyzed by Sanger sequencing to determine the editing status at the target site. Editing efficiency was calculated using the following formula:

## 3 $Editing\;efficiency\;\left(\%\right)\;=\;\left(Number\;of\;edited\;clones/Total\;number\;of\;positive\;clones\right)\;\times \;100\%$Result

### 3.1 Changes in plastid RNA editing efficiency under drought stress

**Figure 1:**
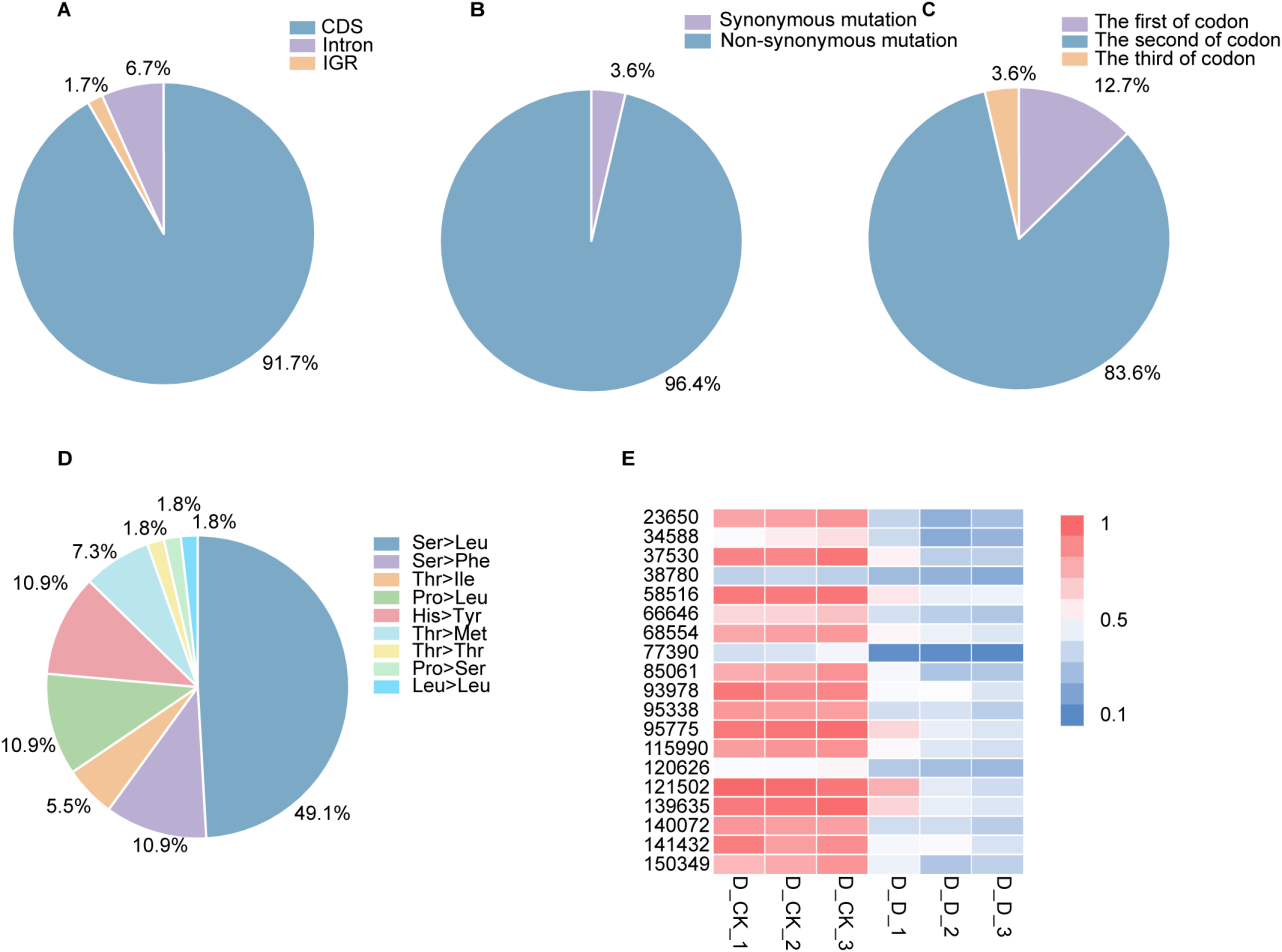
Statistical information on plastid RNA editing sites under drought stress. **(A)** Location of RNA editing sites on the plastid genome. **(B)** Proportion of RNA editing resulting in synonymous and non-synonymous substitutions. **(C)** Location of RNA editing on codons. **(D)** RNA editing results in amino acid changes. **(E)** Plastid genome RNA editing efficiency under drought, the vertical axis represents RNA editing sites; the horizontal axis represents drought control and treatment groups; the displayed bar 0.1–1 indicates RNA editing efficiency.

Under drought stress conditions, 60 RNA editing sites were detected in the plastid genome of Dongfudou 3, with 91.67% located in coding sequences, one site in an intron region, and four sites in intergenic regions (**Figure 1A**). Among the coding sequence sites, 96.36% were nonsynonymous mutations, all of which increased the hydrophobicity of the encoded amino acids; only 3.64% were synonymous mutations (**Figure 1B**). Editing showed a strong preference for the second codon position, which accounted for 46 sites, representing 83.64% of the total. This was followed by the first position, with seven sites (12.73%), while the third position was the least frequent, comprising only two (3.64%) (**Figure 1C**). In terms of amino acid substitutions, serine to leucine was the most common change, observed at 27 sites, representing 49.09% of the total, followed by Ser to Phe, Pro to Leu, and His to Tyr, each accounting for 10.91% (**Figure 1D**). All site information is presented in **Supplementary Table 2**.

All editing efficiency values represent means of three biological replicates, with statistical significance assessed by Kruskal-Wallis test (*P*< 0.05, change ≥ 30%), and it was found that under drought, the efficiency of most sites decreased, yielding a total of 19 significantly fluctuating sites (**Figure 1E**).

### 3.2 Changes in mitochondrial RNA editing efficiency under drought stress

**Figure 2:**
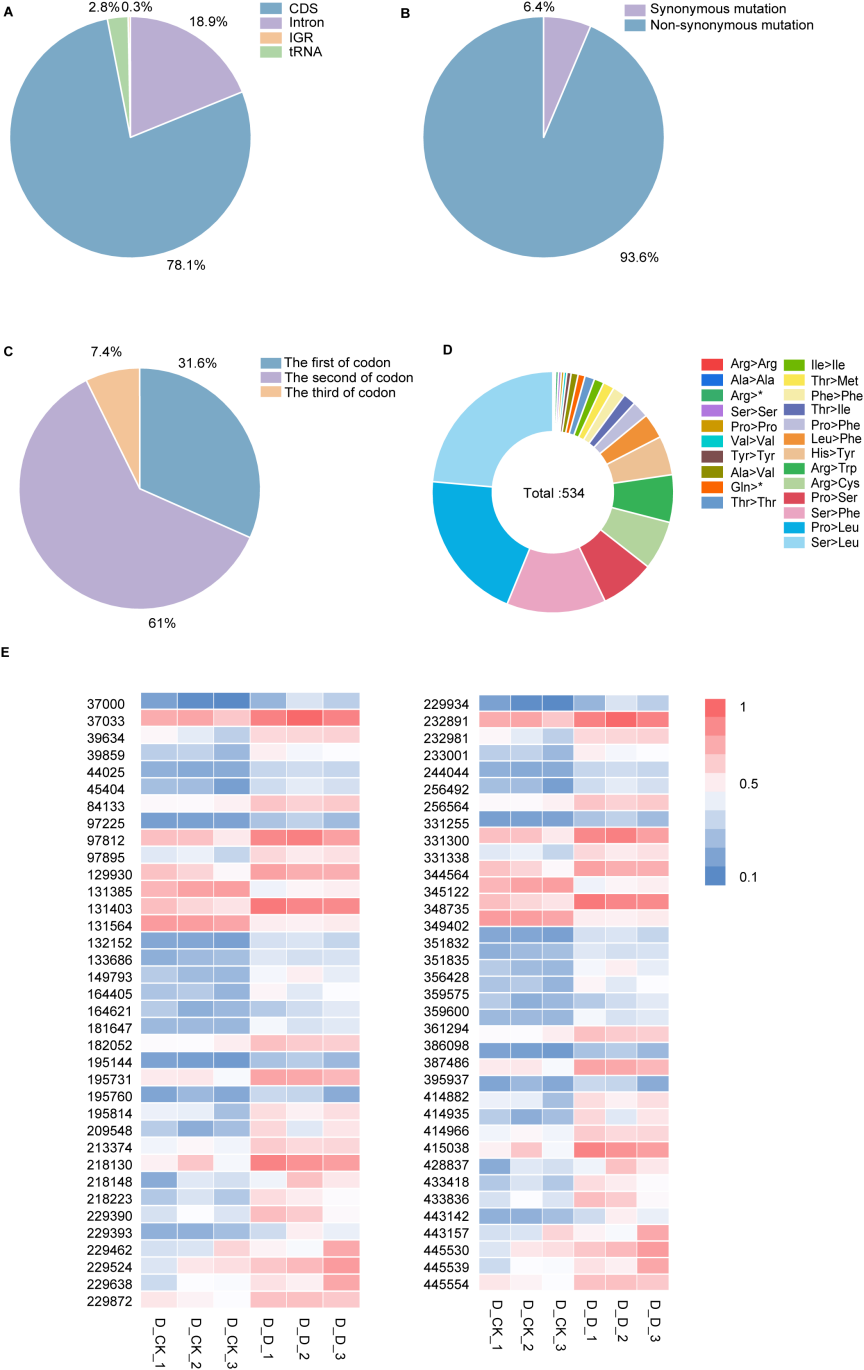
Statistics of mitochondrial RNA editing sites under drought stress. **(A)** Location of RNA editing sites on the mitochondrial genome. **(B)** Location of RNA editing on codons. **(C)** Proportion of RNA editing resulting in synonymous and non-synonymous substitutions. **(D)** RNA editing resulting in amino acid changes. **(E)** RNA editing efficiency of mitochondrial genomes under drought stress, the vertical axis represents RNA editing sites; the horizontal axis represents drought stress control and treatment groups; the displayed bar 0.1–1 indicates RNA editing efficiency.

Under drought stress, a total of 684 RNA editing sites were identified in the mitochondrial genome of Dongfudou 3. Among these sites, 78.07% were located within coding sequences, 18.86% were found in intergenic regions, 2.79% were situated in introns, and a small fraction of 0.29% were detected in tRNA regions (**Figure 2A**). Focusing on the sites within coding sequences, the majority of 93.63% represented nonsynonymous mutations. Notably, an overwhelming 96.40% of these mutations contributed to an increase in the hydrophobicity of the encoded amino acids, while only a minor portion of 3.60% enhanced the hydrophilicity (**Figure 2B**). The editing process demonstrated a clear preference for the second codon position, accounting for 61.05% of the observed events. The first codon position followed with 31.65%, whereas the third codon position was the least frequent, representing only 7.30% of the total (**Figure 2C**). In terms of amino acid substitutions, the most common modification involved the conversion of serine to leucine, which occurred at 23.60% of the editing sites. This was closely followed by the substitution of proline to leucine, observed in 20.22% of cases. Additionally, the conversion of serine to phenylalanine was also prevalent, representing 13.30% of the editing events. These patterns collectively formed the primary modification landscape (**Figure 2D**). All site information is presented in **Supplementary Table 3**.

All editing efficiency values represent means of three biological replicates, with statistical significance assessed by Kruskal-Wallis test (*P*< 0.05, change ≥ 30%), identifying a total of 71 efficiency variant sites, which overall showed an increasing trend (**Figure 2E**).

### 3.3 Evolutionary analysis of the soybean PPR gene family

**Figure 3:**
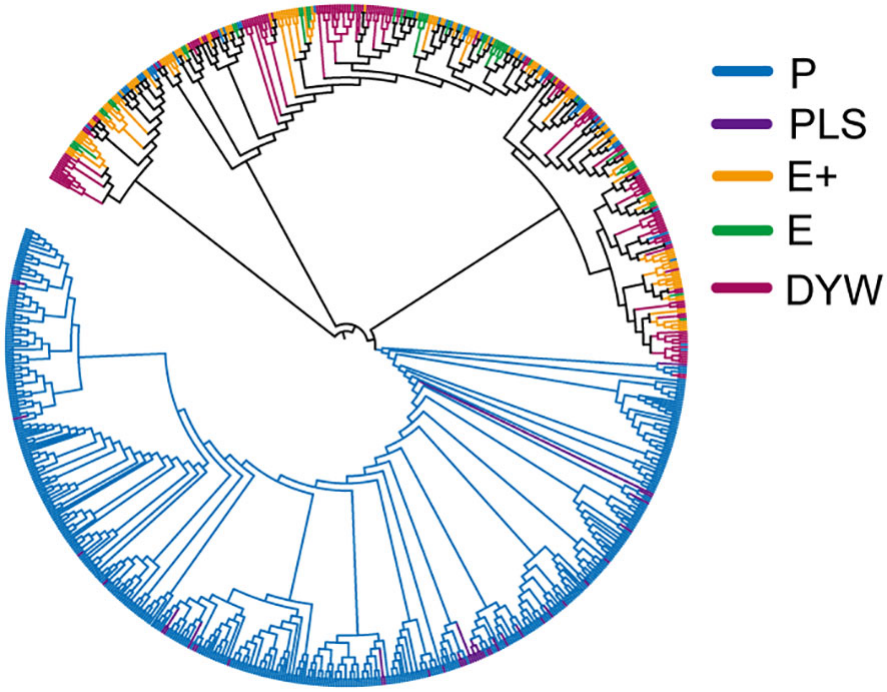
Maximum likelihood tree analysis of the soybean PPR gene family. The figure shows a circular phylogenetic tree of the PPR protein family, in which branches of different colors represent distinct functional subtypes. The branching topology reflects evolutionary relationships, illustrating the phylogenetic connections and population structure of the PPR protein family.

PPR proteins are an important class of RNA editing factors, widely distributed in plants, and are one of the largest families in higher plants (Qin T et al., 2021). Many studies have shown that they play an important role in plant growth and development; therefore, studying the PPR gene family is particularly important for us to understand the phenomenon of RNA editing. Based on the corrected genome file of the Dongfudou 3 from this laboratory, an initial screening was conducted, resulting in a total of 849 candidate soybean PPR protein sequences. After removing redundancy and performing quality assessment, 822 high-confidence members of the soybean PPR gene family were retained. These genes were classified into P class and PLS class. The PLS class was further divided into PLS, E, E+, and DYW subgroups according to their C-terminal domain composition. (**Figure 3**).

Statistical data show that the P-type subfamily constitutes the largest functional group within the soybean PPR gene family, containing 469 member genes, accounting for 57.06% of the total, the highest proportion among all subfamilies; followed by the DYW subfamily, which contains 142 member genes, accounting for 17.27% of the total; the PLS subfamily contains 39 member genes, accounting for 4.74%; the E subfamily and E+ subfamily together contain 172 member genes, accounting for 20.92% of the total. **Supplementary Table 4** displays information on all members.

### 3.4 Analysis of conserved domains and gene structure of soybean DYW-type PPR genes

Researchers generally believe that the PPR motif itself does not have catalytic activity, and the C-to-U (cytidine to uridine) deamination reaction requires the presence of a DYW domain to complete. Therefore, we focus on studying the DYW-type PPR proteins in the PPR family. The CDS region exhibited a relatively compact overall structure, with a continuous coding sequence and minimal or no introns, consistent with the high functional conservation characteristic of PPR protein-coding sequences (**Supplementary Figure 1A**). The CDS is primarily composed of multiple tandem PPR repeat units, each approximately 35 amino acids in length and a C-terminal DYW catalytic domain. These functional modules require precise amino acid sequences to ensure accurate RNA recognition and catalytic activity.

In contrast, the UTR regions displayed greater variability, particularly in the 5′ and 3′ untranslated regions (5′UTR and 3′UTR). In some genes (e.g., *Gm_DFD3_03046.t01*), the UTR length significantly exceeded that of the CDS, reflecting a high degree of sequence divergence. This pronounced variability suggests that the UTRs may play critical roles in the post-transcriptional regulation of PPR protein expression including the modulation of mRNA stability, translation efficiency, and subcellular localization. (**Supplementary Figure 1A**).

Motif analysis of soybean DYW-type PPR proteins revealed a clear modular architecture in their protein structure (**Supplementary Figure 1B**). The N-terminal to central regions were densely populated with multiple conserved motifs (e.g., Motif 1, 5, 9), which correspond to PPR repeat units responsible for specific RNA recognition and binding (**Supplementary Figure 1B**). These motifs exhibited a linear, tandem-repeat arrangement, consistent with the structural organization of canonical PPR domains.

In contrast, the C-terminal region was enriched with motifs associated with catalytic functionality (e.g., Motif 2, 6, 7), which are likely to encompass the DYW triad catalytic domain and are responsible for executing the chemical modification required for RNA editing (i.e., C-to-U conversion) (**Supplementary Figure 1B**).Core motifs (e.g., Motif 1, 5) were highly conserved and positionally stable across different DYW-type PPR proteins, underscoring their essential functional roles within the PPR family. In contrast, protein-specific motifs (e.g., Motif 4, 8) showed variable distribution patterns, reflecting functional diversification among individual PPR members (**Supplementary Figure 1B**). Overall, the structural organization of soybean DYW-type PPR proteins followed a hierarchical modular pattern, characterized by: a recognition module (N-terminal PPR-associated motifs) responsible for RNA binding, and a regulatory/catalytic module (C-terminal key motifs) involved in editing activity. This clear modular and functional compartmentalization provides a structural basis for precise targeting and regulation of RNA editing (**Supplementary Figure 1B**).

### 3.5 Tissue-specific expression analysis of soybean DYW-type PPR genes

**Figure 4:**
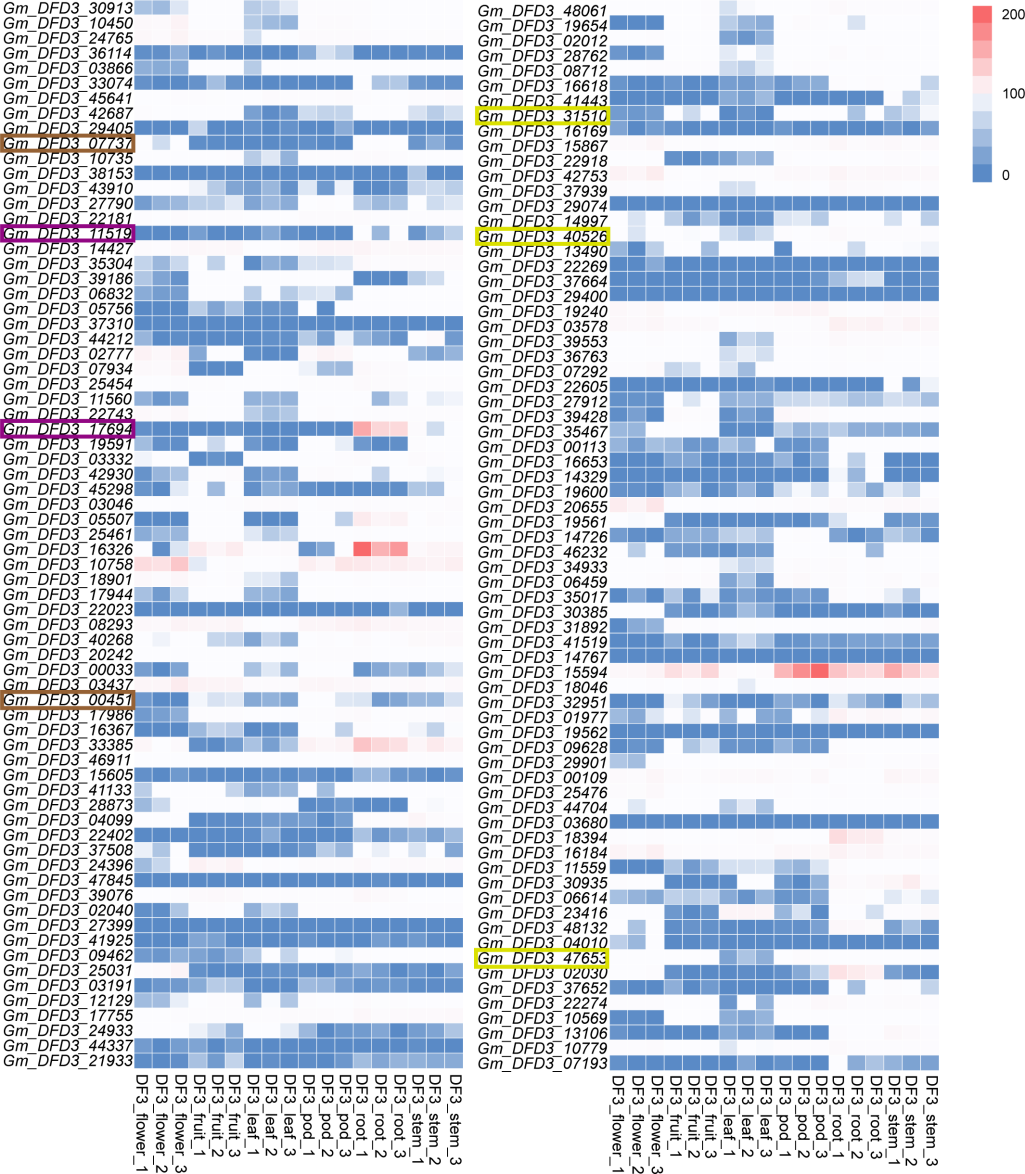
Statistics of DYW-type PPR gene expression levels in different tissue parts. This heatmap shows the TPM expression levels of DYW-type PPR family genes in soybean across flower, fruit, leaf, seed, root, and stem tissues (including biological replicates). The gradient from blue (TPM≈0) to red (TPM≈200) represents the gene expression TPM value from low to high. The left vertical axis shows the PPR gene IDs, and the bottom horizontal axis labels indicate tissue-replicate combinations. The genes in the yellow box in the figure are highly expressed in flowers, pods, and seeds; the genes in the purple box are highly expressed in roots, stems, and leaves; the genes in the brown box are stably expressed in all tissues.

Clustering analysis of the TPM expression levels of the DYW-type PPR gene family across six tissues roots, stems, leaves, flowers, pods, and seeds revealed that certain family members exhibit highly tissue-specific expression patterns. Notably, some genes were specifically highly expressed in reproductive organs such as flowers, pods, and seeds, including *Gm_DFD3_31510* (Wm82_a6 corresponding ID: *Glyma.13G179700*), *Gm_DFD3_40526* (Wm82_a6 corresponding ID: *Glyma.17G099500*), and *Gm_DFD3_47653* (Wm82_a6 corresponding ID: *Glyma.20G112100*), suggesting their potential key roles in seed development and embryogenesis (**Figure 4**). Conversely, other genes showed relatively higher expression in vegetative organs such as roots, stems, and leaves, exemplified by *Gm_DFD3_11519* (Wm82_a6 corresponding ID: *Glyma.05G240300*), and *Gm_DFD3_17694* (Wm82_a6 corresponding ID: *Glyma.08G086500*), indicating their involvement in the growth regulation of these organs (**Figure 4**). Additionally, a few genes, such as *Gm_DFD3_07737* (Wm82_a6 corresponding ID: *Glyma.04G057300*) and *Gm_DFD3_00451* (Wm82_a6 corresponding ID: *Glyma.01G048100*), exhibited moderate expression levels across multiple tissues, possibly serving fundamental regulatory functions (**Figure 4**). Of particular interest is the overall higher magnitude of gene expression in vegetative tissues compared to reproductive tissues, with the most pronounced expression observed in leaves. This expression pattern supports the relevance of *Gm_DFD3_00451* to leaf plastid responses under drought, whereas soybean hairy roots were used as a rapid system for preliminary functional validation.

### 3.6 Analysis of PPR protein RNA target recognition sites

**Figure 5:**
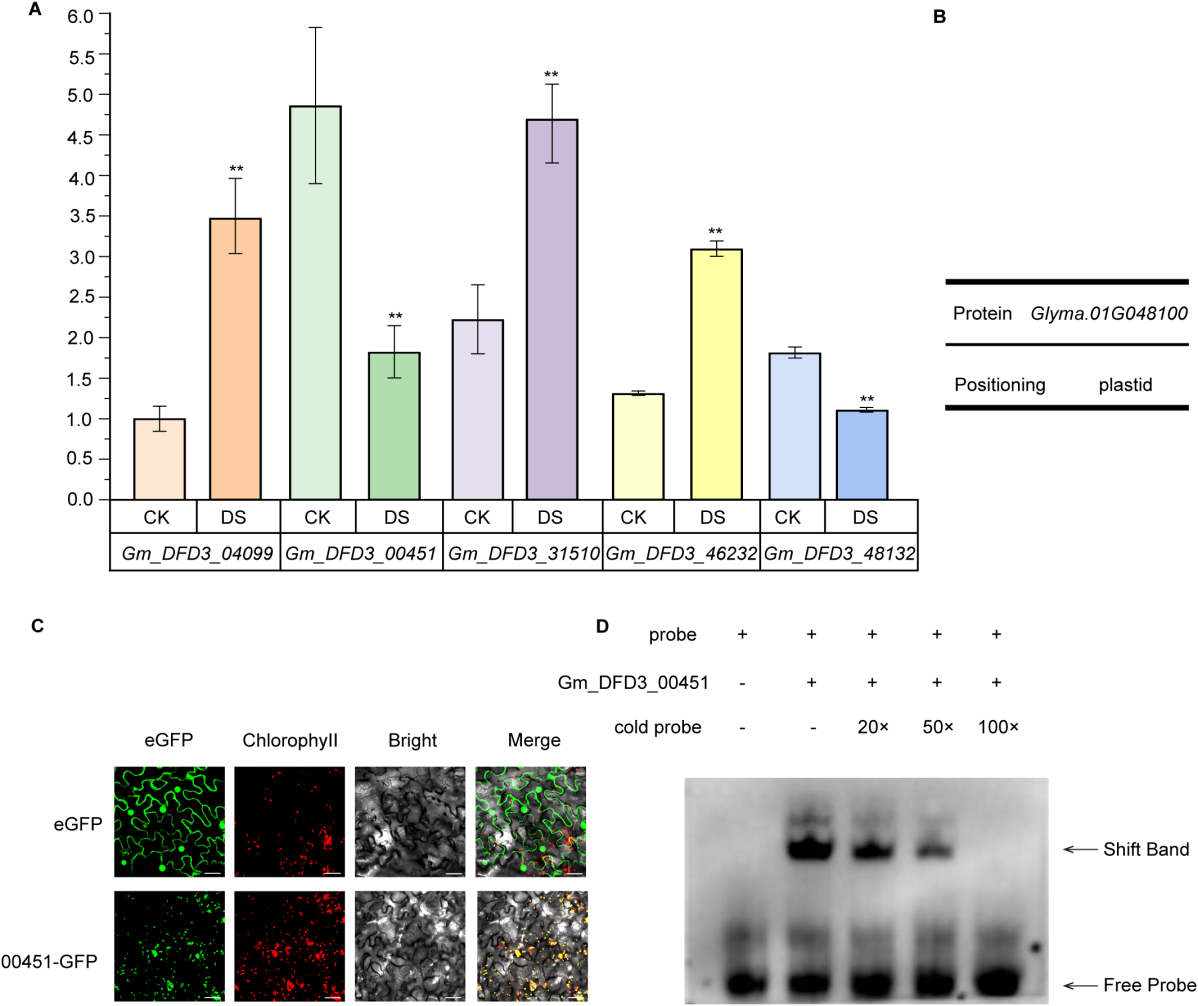
Quantitative analysis of differential gene expression in PPR and subcellular localization of Gm_DFD3_00451 and RNA EMSA results. **(A)** CK and DS represent the status of the gene under normal water conditions and drought stress, respectively, the data were statistically analyzed using an independent sample t-test, where ‘**’ indicates a highly significant difference (*P*< 0.01). **(B)** Subcellular localization results of the protein. **(C)** In the figure, eGFP is the empty vector control, 00451-GFP is the target protein fused with a GFP tag, 00451 is the abbreviation for the gene Gm_DFD3_00451 and the scale bar in the figure is 50 μm. **(D)** “+” indicates the addition of this component, “-” indicates not added; 20×, 50×, 100× respectively represent non-biotin-labeled probe/biotin-labeled probe at ratios of 20/1, 50/1, 100/1.

Based on the preliminary transcriptomic sequencing results under drought stress, five candidate genes with pronounced tissue-specific expression were selected from the differentially expressed members of the PPR gene family. Their expression levels were precisely quantified using qRT-PCR to elucidate their potential functions in drought response (**Figure 5A**), under drought stress, the expression of these five candidate genes significantly differed from the control group. The trend of quantitative experimental data is consistent with the transcriptome results.

Specifically, *Gm_DFD3_00451* (Wm82_a6 corresponding ID: *Glyma.01G048100*) exhibited relatively high expression under normal water conditions, but its expression significantly decreased following drought stress. *Gm_DFD3_31510* (Wm82_a6 corresponding ID: *Glyma.13G179700*) showed the highest expression after drought stress. In contrast, *Gm_DFD3_46232* (Wm82_a6 corresponding ID: *Glyma.19G202500*) and *Gm_DFD3_04099* (Wm82_a6 corresponding ID: *Glyma.02G227300*) displayed relatively lower expression levels; however, their expression also increased under drought stress. These findings suggest that all five genes are shown drought-responsive expression patterns. Notably, *Gm_DFD3_00451* exhibited a substantial response magnitude under drought stress, indicating its pivotal role in the response process (**Figure 5A**). Consequently, selecting this gene for subsequent studies may facilitate the identification of core regulatory mechanisms.

The PPRCODE server was used to annotate the repeat architecture of the PPR proteins. Gm_DFD3_00451 was identified as a drought-responsive gene (**Figure 5A**). Its encoded protein was predicted to contain ten distinct PPR repeat units (**Table 1**). *Gm_DFD3_00451* was identified as the most promising candidate gene. This gene presented the most remarkable expression reduction under drought treatment with log_2_FC=-3.1 (**Supplementary Table 5**). It obtained a high confidence score of 13.35 in the prediction targeting *ndhD*-878. The transcript abundance in leaf tissue stood at TPM = 23.45 and subcellular localization analysis confirmed its distribution in plastid. *Gm_DFD3_31510* exhibited the most significant expression increase with log_2_FC=2.4 (**Supplementary Table 5**). Yet its expression level remained extremely low in leaf tissue. This gene mainly expressed in floral organs, which did not match the functional characteristics of leaf chloroplast responding to drought stress. Other candidate genes failed to meet screening criteria due to insufficient prediction reliability, incorrect subcellular localization or insignificant expression variation under drought conditions. *Gm_DFD3_00451* was ultimately chosen for subsequent systematic functional verification (**Supplementary Table 5**).

**Table 1:** Analysis of repeated sequences in Gm_DFD3_00451 protein.

Start	End	Sequence	#5	#35	PPR Code	RNA base	Length	Score
100	134	IVLFNTMARGYARFDDPLRAILLCSQVLCSGLLPD	N	D	ND	A>G	35	8.21
135	169	DYTFSSLLKACARLKALEEGKQLHCLAVKLGVGDN	S	N	SN	G>>C	35	9.032
170	200	MYVCPTLINMYTACNDVDAARRVFDKIGEPC	P	C	PC	C	35	7.772
201	235	VVAYNAIITSCARNSRPNEALALFRELQESGLKPT	N	T	NT	U>>C	35	9.394
236	270	DVTMLVALSSCALLGALDLGRWIHEYVKKNGFDQY	L	Y	NN	U>C	35	13.351
271	301	VKVNTALIDMYAKCGSLDDAVSVFKDMPRRD	T	D	TD	C>U>G	35	10.742
302	336	TQAWSAMIVAYATHGHGSQAISMLREMKKAKVQPD	S	D	SD	A>U	35	12.145
337	372	EITFLGILYACSHTGLVEEGYEYFHSMTHEYGIVPS	L	S	LS	A>>G	35	11.509
373	407	IKHYGCMIDLLGRAGRLEEACKFIDELPIKPTPIL	G	L	GL	C	35	12.342
439	473	GGDYVILSNLCARNGRWDDVNHLRKMMVDKGALKV	V	V	VV	U>C>G	35	8.396

In the table, the combined diresidues in the PPR gene repeat sequences are located at positions 5 and 35 (corresponding to residues 6′ and 1′in Barkan), as well as their corresponding RNA editing targets.

By analyzing the correspondence patterns between the PPR code and RNA bases, we identified the potential Analysis of the PPR code-RNA base correspondence revealing the putative RNA target sequence recognized by Gm_DFD3_00451 (**Figure 5B**). Utilizing TBtools software, the DFD3 chloroplast DNA genome file was converted into an RNA genome file. Setting an e-value threshold of 0.01, the RNA target sequence was aligned against the DFD3 chloroplast RNA genome. Integrating the PPRCODE prediction with drought-responsive editing sites revealed that Gm_DFD3_00451 was a candidate regulator of the plastid *ndhD*-878 editing site, which showed a marked change in editing efficiency under drought stress.

The subcellular localization of Gm_DFD3_00451 in the chloroplast was predicted by both LOCALIZER and DeepLoc-2.1. To experimentally verify this prediction, a fusion expression vector pBWA(V)HS-gfp-48100, combining *Gm_DFD3_00451* with GFP, was constructed. Confocal microscopy of transgenic tobacco leaves revealed a high degree of overlap between the fluorescence signals of the experimental group and the chloroplast’s autofluorescence, confirming the chloroplastic localization of the protein (**Figure 5C**).

Cold-probe competition reduced the shifted signal, supporting a competitive interaction between Gm_DFD3_00451 and the *ndhD*-878 upstream RNA probe. Because mutated and unrelated probes were not included, the sequence specificity of this interaction requires further validation. (**Figure 5D**).

### 3.7 Analysis of the effect of Gm_DFD3_00451 protein on *ndhD*-878 site editing efficiency under stress

**Figure 6:**
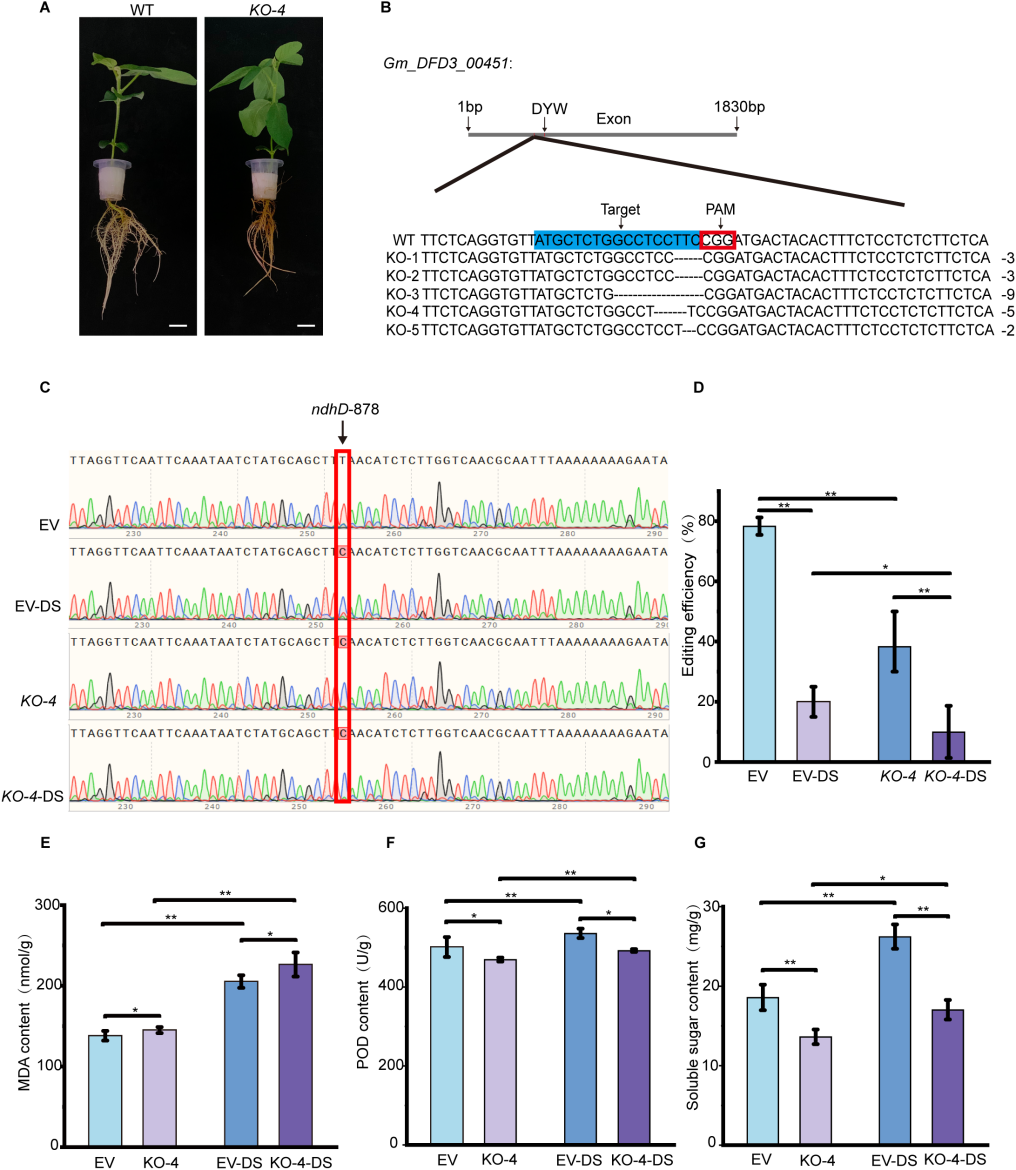
Candidate gene function validation and RNA editing efficiency analysis. **(A)** Display of gene-edited lines, scale bar in the figure is 0.5 cm, WT: wild type; KO-4: knockout line. **(B)** Schematic diagram of base changes in gene editing, showing the target sequence of sgRNA and the location of the PAM site, WT: wild type; KO-1 to KO-5: knockout lines, with the number of deleted bases indicated after each sequence. **(C)** EV and KO-4 represent the unloaded plants and knockout plants under normal moisture conditions, respectively; EV-DS and KO-4-DS represent the unloaded plants and knockout plants under drought stress conditions, respectively. The red box shows changes in the editing efficiency at the *ndhD*-878 site in different plants. **(D)** EV and KO-4 represent empty vector plants and knockout plants under normal water conditions, respectively; EV-DS and KO-4-DS represent empty vector plants and knockout plants under drought stress conditions, respectively. Data analysis was performed using an independent samples T-test. * Indicates a significant difference between the normal water group and the drought stress group (*P*< 0.05), ** indicates a highly significant difference between the two groups (*P*< 0.01). **(E)** Changes in soybean MDA content under drought stress **(F)** Changes in soybean POD content under drought stress. **(G)** Changes in soybean soluble sugar content under drought stress; EV and KO-4 are empty vector plants and knockout plants under normal water conditions; EV-DS and KO-4-DS are empty vector plants and knockout plants under drought stress. The study results were statistically analyzed using an independent sample T-test. The specific significance marking rules are as follows: * indicates a significant difference between comparisons (*P*< 0.05), ** indicates a highly significant difference between comparisons (*P*< 0.01).

The CRISPR/Cas9-mediated gene editing construct targeting Gm_DFD3_00451 was introduced into K599 for hairy root-mediated transformation (**Figure 6A**).

To assess gene editing efficiency and editing patterns, genomic DNA was extracted from two positive hairy roots per line from five independent gene−edited soybean hairy root lines and an empty vector control, followed by amplification of the target site to detect potential mutations. Sanger sequencing confirmed the presence of deletions at the target site, indicating successful editing (**Figure 6B**). PCR-based genotyping results are shown in **Supplementary Figure 2A**.

To assess the impact of *Gm_DFD3_00451* gene editing on the RNA editing efficiency at the *ndhD*-878 site under drought stress conditions, we selected the knockout line KO-4, which exhibited the highest frequency of frameshift mutations, as the experimental material. Specific primers covering a 300 bp region upstream and downstream of the RNA editing site were designed. Using KO-4 cDNA as the template, we performed PCR amplification of the target fragment. Sanger sequencing of the purified PCR product further verified the occurrence of C-to-U RNA editing at the *ndhD*-878 site (**Figure 6C**). PCR validation results are shown in **Supplementary Figure 2B**.

Single-colony Sanger sequencing of the cloned PCR products spanning the *ndhD*-878 site revealed distinct changes in RNA editing efficiency among treatments (**Figure 6D**). Under well-watered conditions, editing efficiency in KO-4 was significantly lower than in EV, indicating that knockout of the *Gm_DFD3_00451* reduced the basal editing level at this site. Under drought stress, both EV-DS and KO-4-DS showed a marked decline in editing efficiency to their well-watered counterparts. Notably, editing efficiency in KO-4-DS dropped to 10%, indicating that drought stress mild suppresses *ndhD*-878 editing and that the knockout of *Gm_DFD3_00451* further exacerbates this inhibitory (**Figure 6D**).

Under well-watered conditions, the MDA content in KO-4 was significantly higher than in EV. Drought stress markedly increased MDA levels in both genotypes, with KO-4-DS consistently showing significantly higher MDA content than EV-DS under stress. Notably, the drought-induced increase was greater in the knockout line than in the EV control. These results indicate that knockout of *Gm_DFD3_00451* exacerbates drought-induced membrane lipid peroxidation, suggesting a reduced capacity for drought tolerance (**Figure 6E**). Under well-watered conditions, POD activity in KO-4 was significantly lower than in EV. Drought stress significantly induced POD activity in both genotypes, but the increase in KO-4-DS was markedly smaller than that in EV-DS. Consequently, under drought stress, POD activity in KO-4-DS remained significantly lower than in EV-DS. These results indicate that knockout of *Gm_DFD3_00451* impairs POD accumulation under drought stress (**Figure 6F**). Under well-watered conditions, soluble sugar content in KO-4 was significantly lower than in EV. Drought stress significantly induced soluble sugar accumulation in both genotypes, but the increase in KO-4-DS was markedly smaller than that in EV-DS. Consequently, soluble sugar content in KO-4-DS remained significantly lower than in EV-DS under stress. These results indicate that knockout of *Gm_DFD3_00451* impairs drought-induced soluble sugar accumulation (**Figure 6G**).

## 4 Discussion

### 4.1 RNA editing of organelle genomes under drought stress

In flowering plants, plastid genomes typically harbor 20–60 RNA editing sites, whereas mitochondrial genomes contain 300–600 sites (Ichinose, M. and Sugita, M. 2016). In tall fescue, 60 plastid editing sites supported by RNA-Seq have been identified (Wang M et al., 2016). Here, we systematically profiled organellar RNA editing in drought-stressed Dongfudou 3 and identified 60 plastid and 684 mitochondrial editing sites, consistent with the conserved range reported for angiosperms.

Organellar RNA editing in soybean exhibited conserved genomic distribution patterns. Consistent with the full transcription of organellar genomes (Shi C et al., 2016), we included non-coding regions in our analysis. Most editing events occurred in coding sequences (CDSs), 91.67% in plastids and 78.07% in mitochondria. The lower proportion of CDS-localized edits in mitochondria reflects their sparse gene arrangement and larger intergenic regions. Notably, tRNA editing was detected in soybean mitochondria, mirroring observations in Selaginella (Grewe F et al., 2011). A subset of editing sites was detected in intronic regions, indicating that organellar RNA editing is not limited to CDS correction. These intronic editing events may affect local RNA structure and potentially influence intron splicing, but their functional relevance requires further validation. Similar observations have been reported in other plant mitochondrial genomes; for example, the *Lycopodium japonicum* plant mitochondrial genome contains abundant group II introns and hundreds of RNA editing sites, illustrating the close association between mitochondrial genome organization and RNA editing diversity (Sun et al., 2024).

C-to-U conversion was the predominant editing type, and most CDS edits resulted in nonsynonymous substitutions that increased amino acid hydrophobicity. Editing preferentially targeted the second codon position, followed by the first, while edits at the third position were rare pattern consistent with the tendency to alter protein function. Third-position edits were mostly synonymous and had minimal impact on protein structure, whereas substitutions at the first and second positions readily modified amino acid physicochemical properties. Synonymous editing may affect mRNA secondary structure and splicing, and third-position edits may represent “accidental editing” (Edera AA et al., 2018).

Amino acid changes induced by organellar RNA editing were functionally biased. Ser→Leu substitutions were most frequent, increasing hydrophobicity at subunit interfaces and thereby promoting protein complex stability. Pro→Leu substitutions were also common, alleviating proline-mediated α-helix disruption. Edited residues were enriched in protein helices, interaction interfaces, and structural cores, indicating that RNA editing modulates key structural domains to enhance protein stability and optimize conformation (Yura and Go, 2008).

Under drought stress, plastid RNA editing efficiency generally decreased, whereas mitochondrial editing efficiency significantly increased. This divergence may reflect, at least in part, the distinct functional roles of plastids and mitochondria under drought stress. As the primary site of photosynthesis, plastid activity is suppressed under drought, and RNA editing, as a critical post-transcriptional regulatory mechanism, is accordingly downregulated. Reduced editing at the *ndhD*-878 site impairs the function of the plastid NDH complex, disrupting cyclic electron flow and redox homeostasis, which in turn promotes the accumulation of ROS (Qiao et al., 2024). Excess ROS accelerates membrane lipid peroxidation, leading to elevated MDA content, and inhibits the synthesis and activity of antioxidant enzymes, reducing POD activity and weakening the intrinsic ROS scavenging system (Wang et al., 2025). Disrupted chloroplast energy metabolism further restricts photosynthetic carbon fixation and assimilates transport, altering soluble sugar accumulation and distribution in leaves (Du et al., 2020). Collectively, the overall decrease in plastid editing may represent part of the plastid post-transcriptional response to water deficit, although its adaptive significance requires further validation.

In contrast, mitochondria serve as the core of cellular energy metabolism and must maintain basal ATP production under drought. Increased mitochondrial RNA editing efficiency optimizes the function of respiratory chain proteins, ensuring ATP synthesis and providing energy to support drought tolerance (Leilei Peng et al., 2025).

Functional deficiency of *Gm_DFD3_00451* further aggravated the decrease in plastid editing efficiency, accompanied by increased MDA content, enhanced membrane lipid peroxidation, reduced POD activity, impaired ROS scavenging, and altered soluble sugar accumulation, which weakened osmotic adjustment. These physiological changes are tightly associated with the regulation of plastid RNA editing by the candidate gene, confirming its core role in the soybean drought response.

This study has a potential limitation that organellar RNA editing was profiled in leaf tissue, whereas gene function was validated using soybean hairy root transformation. Given the tissue-specific nature of organellar RNA editing and PPR-mediated regulatory networks, results from hairy roots may not fully recapitulate physiological processes in leaves. Future studies using stable transgenic soybean lines and leaf-tissue validation are required to refine the drought resistance regulatory pathway mediated by Gm_DFD3_00451.

### 4.2 Conserved functions and regulatory mechanisms of PPR proteins in plant abiotic stress responses

Plastids and mitochondria, as semi-autonomous organelles, perceive environmental stress signals and mediate adaptive responses, making organelle-localized PPR proteins key regulators of plant abiotic stress tolerance (Cao Jinzhe et al., 2023; Li Xiulan and Jiang Yueshui., 2018; Lurin C et al., 2004). In *Arabidopsis thaliana*, more than ten PPR proteins are drought-responsive. The chloroplast PPR protein RARE1 mediates *accD* C794 editing to enhance thermotolerance (Huang C et al., 2023); SVR7 regulates oxidative stress responses, and its mutants accumulate higher ROS levels and exhibit reduced tolerance to photoinhibition (Lv et al., 2014); GUN1 maintains oxidative homeostasis and basal thermotolerance via plastid-nucleus communication (Cottage A et al., 2010; Lasorella C et al., 2022). Several mitochondria-localized PPRs, including PPR40, ABO5, and ABO8, also function in drought responses, while the dual-targeted SOAR1 positively regulates abiotic stress tolerance and ABA signaling (Jiang SC et al., 2015).

Similar observations have been reported in crops. Genome-wide analysis in rice identified 491 PPR genes, many of which showed stress-responsive expression, including 73 genes upregulated under drought (Chen G et al., 2018). Functional studies have also shown that rice PPR proteins can influence chloroplast development, ABA or salt responses and mitochondrial function, as illustrated by OsV4 (Gong et al., 2014), TCD10 (Wu et al., 2016), WSL (Wang Y et al., 2017), PPS1 (Xiao et al., 2018), and OSNBL3 (Qiu T et al., 2021). These studies suggest that PPR proteins participate broadly in organellar RNA metabolism and stress-associated development in crops, although their specific molecular targets and stress functions differ among family members.

Chloroplast C-to-U RNA editing relies on coordinated actions of multiple enzymes and cofactors to form a complex regulatory network. The DYW domain acts as the core catalytic module, with a conserved gated zinc-shutter structure that maintains an autoinhibited state and may become activated during editosome assembly, ensuring precise deamination (Takenaka M et al., 2021). In maize, loss-of-function of the DYW-type PPR gene ZmPPR26 abolishes editing at chloroplast *atpA*-1148, disrupts amino acid hydrophobicity transitions, reduces ATP synthase abundance, and impairs chloroplast development (Liu XY et al., 2021). Another PPR-DYW protein, Dek570-1, dually targets mitochondria and chloroplasts; its mutation disturbs multiple mitochondrial editing sites, reduces chloroplast *rpL20–*308 editing efficiency, disrupts plastid gene expression, decreases thylakoid content, and suppresses photosynthesis (Li W et al., 2025). These findings demonstrate that PPR-DYW proteins coordinately maintain functional homeostasis of both mitochondria and chloroplasts.

In this study, we characterized drought-responsive organellar RNA editing in soybean and identified Gm_DFD3_00451 as a plastid-localized DYW-type PPR candidate that may participate in the regulation of *ndhD*-878 editing and drought-associated physiological responses.

Notably, the direct interaction between Gm_DFD3_00451 and *ndhD*-878 requires further validation. Our RNA EMSA assay only included free probe and cold-probe competition controls, lacking protein gradients, mutated/unrelated probes, and biological/technical replicates. Independent experiments are therefore needed to confirm their direct and specific binding.

In summary, this work suggests that Gm_DFD3_00451-mediated *ndhD*-878 editing may represent a link between organellar post-transcriptional regulation and drought adaptation in soybeans. Our findings advance the understanding of organellar RNA editing in crop stress responses and provide a candidate gene for further functional evaluation in soybean drought response.

## Data Availability

The datasets presented in this study can be found in online repositories. The names of the repository/repositories and accession number(s) can be found below: https://www.ncbi.nlm.nih.gov/, PRJNA1463902. https://www.ncbi.nlm.nih.gov/, PRJNA1464061.
