# Supplementary material for: Integrated analysis of organelle RNA editing and DYW- type PPR genes identifies a candidate regulator of plastid ndhD-878 editing under drought stress in soybean
Source: Front Plant Sci. 2026 Jul 6;17:1879625. doi: 10.3389/fpls.2026.1879625 (PMC13381635; doi:10.3389/fpls.2026.1879625)
Supplement: Supplementary file 1 [file Table1.docx]

Supplementary Method

# Identification of the Soybean PPR Gene Family and Phylogenetic Analysis

# To identify high-quality PPR protein sequences in soybean, a local HMMER search was performed against the Dongfudou 3 genome using the PPR family model (PF01535) from Pfam v32.0 (Qin T et al., 2021). An E-value threshold of <1e^-10^ was applied to select candidate PPR proteins (Finn RD et al., 2006). The resulting candidates were further refined by removing redundant sequences and validating domain architectures. A refined set of high-confidence soybean PPR gene sequences was thus obtained. These sequences were subjected to multiple sequence alignment, and a maximum likelihood (ML) phylogenetic tree was constructed using IQ-TREE(V7) with automatic model selection and 1,000 bootstrap replicates (Tamura K et al., 2011). The phylogeny was visualized using iTOL online platform, with branches color-coded according to PPR subfamily classification to infer evolutionary relationships and putative functional diversification within the family.

# Gene Structure, conserved motif and chromosomal distribution analysis of DYW-type PPR members

# Based on the whole-genome annotation of Dongfudou 3, the gene sequences of DYW-type PPR family members were imported into Tbtools (Chen C et al., 2023) to visualize their transcript structures. This visualization displayed the 5′ untranslated region (5′ UTR), CDS, 3′ untranslated region (3′ UTR), and exon-intron architecture, allowing assessment of gene integrity and the potential for alternative splicing events.

# Conserved motifs in the amino acid sequences of DYW-type PPR proteins were predicted using the MEME Suite online analysis platform (https://meme-suite.org/meme/), with the number of motifs set to 10 and all other parameters kept at default values. The chromosomal distribution of DYW-type PPR genes was mapped using the genome visualization module of Tbtools (Chen C et al., 2023).

# Sequential Gene Expression Specificity Analysis

# Transcriptomic data from various Dongfudou 3 tissues were used to obtain TPM (Transcripts Per Million) expression values for DYW‑type PPR genes. As TPM values are normalized for both gene length and sequencing depth, they allow direct comparison of transcript abundance across samples. The resulting expression matrix was imported into R, and a heatmap was generated using the pheatmap package, with color intensity reflecting relative expression levels. Hierarchical clustering analysis was performed alongside heatmap visualization to reveal similarities and differences in expression profiles among family members.

# Subcellular localization

# For subcellular localization analysis, leaves of Nicotiana benthamiana were used. The full-length CDS of *Gm_DFD3_00451* (corresponding to Wm82_a6 ID: *Glyma.01G048100*) was amplified using primer 51CDS-F/R (Supplementary Table 1) and clone into the pBWA(V)HS-GFP vector, for specific experimental methods, refer to Collings DA (Collings DA. 2013).

# Physiological indicators measurement of knockout lines

# Malondialdehyde (MDA) content was determined using the thiobarbituric acid (TBA) colorimetric method (Heath RL and Packer L. 1968). Peroxidase (POD) activity was evaluated by monitoring absorbance changes at 420 nm (Bates L S et al., 1973). Soluble sugar content was measured with the anthrone colorimetric method at 620 nm (YEMM EW and WILLIS AJ. 1954).

# Reference

1. Qin T, Zhao P, Sun J, Zhao Y, Zhang Y, Yang Q, Wang W, Chen Z, Mai T, Zou Y, Liu G and Hao W (2021) Research Progress of PPR Proteins in RNA Editing, Stress Response, Plant Growth and Development. Front. Genet. 10.3389/fgene.2021.765580
2. Finn RD, Mistry J, Schuster-Böckler B, Griffiths-Jones S, Hollich V, Lassmann T, Moxon S, Marshall M, Khanna A, Durbin R, Eddy SR, Sonnhammer EL, Bateman A. (2006) Pfam: clans, web tools and services. Nucleic Acids Res. 10.1093/nar/gkj149.
3. Tamura K, Peterson D, Peterson N, Stecher G, Nei M, Kumar S. (2011) MEGA5: molecular evolutionary genetics analysis using maximum likelihood, evolutionary distance, and maximum parsimony methods. Mol Biol Evol. 10.1093/molbev/msr121.
4. Chen C, Wu Y, Li J, Wang X, Zeng Z, Xu J, Liu Y, Feng J, Chen H, He Y, Xia R. (2023) TBtools-II: A "one for all, all for one" bioinformatics platform for biological big-data mining. Mol Plant. 10.1016/j.molp.2023.09.010.
5. Collings DA. (2013) Subcellular localization of transiently expressed fluorescent fusion proteins. Methods Mol Biol. 10.1007/978-1-62703-613-9_16.
6. Heath RL, Packer L. (1968) Photoperoxidation in isolated chloroplasts. I. Kinetics and stoichiometry of fatty acid peroxidation. Arch Biochem Biophys. 10.1016/0003-9861(68)90654-1.
7. Bates L S, Waldren R P, Teare I D. (1973) Rapid Determination of Free Proline for Water-Stress Studies[J]. Plant and Soil. 10.1007/BF00018060.
8. YEMM EW, WILLIS AJ. (1954) The estimation of carbohydrates in plant extracts by anthrone. Biochem J. 10.1042/bj0570508.
