# Supplementary material for: Integrated analysis of organelle RNA editing and DYW- type PPR genes identifies a candidate regulator of plastid ndhD-878 editing under drought stress in soybean
Source: Front Plant Sci. 2026 Jul 6;17:1879625. doi: 10.3389/fpls.2026.1879625 (PMC13381635; doi:10.3389/fpls.2026.1879625)
Supplement: Supplementary file 2 [file Table2.docx]

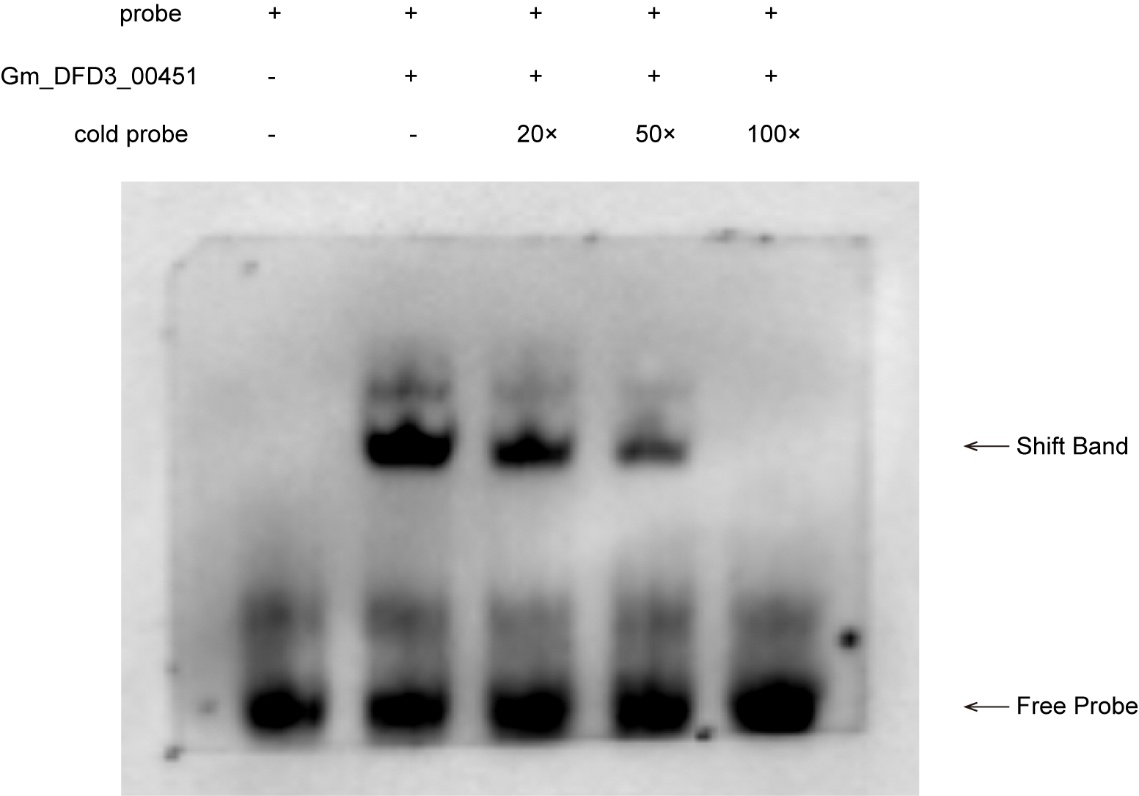


**Figure.5D** "+" indicates the addition of this component, "-" indicates not added; 20×, 50×, 100× respectively represent non-biotin-labeled probe/biotin-labeled probe at ratios of 20/1, 50/1, 100/1.


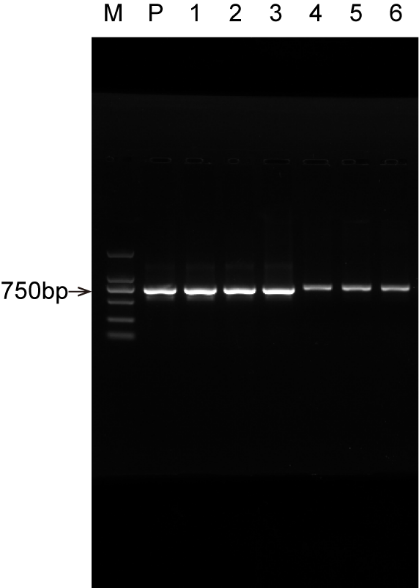


**Supplementary Figure 2A** PCR verification of knockout positive roots, M: DNA marker 2K Plus II; P: PCR verification of empty vector plant roots; 1-6: PCR verification of knockout positive roots. The samples in lanes 1-5 shown in the text are correctly sequenced knockout samples.
