# Supplementary material for: Integrated analysis of organelle RNA editing and DYW- type PPR genes identifies a candidate regulator of plastid ndhD-878 editing under drought stress in soybean
Source: Front Plant Sci. 2026 Jul 6;17:1879625. doi: 10.3389/fpls.2026.1879625 (PMC13381635; doi:10.3389/fpls.2026.1879625)
Supplement: Supplementary file 3 [file Table3.docx]

***Supplementary Material***

1. **Supplementary Figures and Tables**
   1. **Supplementary Tables**

**Supplementary Table 1 Complete Primer Sequence Information.**

|  | Primer Name | Primer sequence |
| --- | --- | --- |
| Quantitative Primer Information | *Gm_DFD3_04099*-F | TCGACGGATGCAGTTTTCCA |
|  | *Gm_DFD3_04099*-R | TCTGCAGGCAGTCATCAAGG |
|  | *Gm_DFD3_00451*-F | ACACCCATTCTCCAATGCGT |
|  | *Gm_DFD3_00451*-R | TCGTTGCAGGCAGTGTACAT |
|  | *Gm_DFD3_31510*-F | ATGGACGGAAAAGAAAGG |
|  | *Gm_DFD3_31510*-R | CTTGTTCAGATTATTGGTGA |
|  | *Gm_DFD3_46232*-F | CTCCTCTCTCGCATCGTTCC |
|  | *Gm_DFD3_46232*-R | AAAAACCGCACGTGCTCTTC |
|  | *Gm_DFD3_48132*-F | TGCCTTTCAAGCCGGATTCT |
|  | *Gm_DFD3_48132*-R | TACATGAACACTGGCCGTCC |
|  | Primer Name | Primer sequence |
| Information on Cloning Primers | 51CDS-F | ATGGCAACAACACCCATTCTCCAA |
|  | 51CDS-R | GAAATGCTCCTGTGGGGATTACTGG |
|  | Protein Name | Primer sequence |
| RNA EMSA Probe Sequences | Gm_DFD3_00451 | GCUUCAACAUCUCUUGGUCAACGCAAUUUA |
|  | Primer Name | Primer sequence |
| Knockout validation primers | 00451-Cas-F | GCAGACATAGAGGATGAAGAGAAAG |
|  | 00451-Cas-R | GTAAAAACAATAATTGTATGGTTGA |

**Supplementary Table 2 Statistics of all plastids editing sites under drought stress.**

| Chromosomal location | Gene name | Genotype | Position on the gene | Base change | Codon change | Codon change position | Protein changes | Protein changes | Synonymous/Non-synonymous Mutation |
| --- | --- | --- | --- | --- | --- | --- | --- | --- | --- |
| 10778 | *ndhC* | exon | 40 | C>U | CUA>UUA | 1 | Leu>Leu | - | Synonymous |
| 23650 | *rps14* | exon | 80 | C>U | UCA>UUA | 2 | Ser>Leu | hydrophobic | Non-synonymous |
| 25366 | *IGR* | IGR | - | G>A | - | - | - | - | - |
| 34588 | *rpoB* | exon | 338 | C>U | UCU>UUU | 2 | Ser>Phe | hydrophobic | Non-synonymous |
| 34801 | *rpoB* | exon | 551 | C>U | UCA>UUA | 2 | Ser>Leu | hydrophobic | Non-synonymous |
| 34816 | *rpoB* | exon | 566 | C>U | UCG>UUG | 2 | Ser>Leu | hydrophobic | Non-synonymous |
| 36250 | *rpoB* | exon | 2000 | C>U | UCU>UUU | 2 | Ser>Phe | hydrophobic | Non-synonymous |
| 37530 | *rpoC1* | exon | 41 | C>U | UCA>UUA | 2 | Ser>Leu | hydrophobic | Non-synonymous |
| 38780 | *rpoC1* | exon | 488 | C>U | UCA>UUA | 2 | Ser>Leu | hydrophobic | Non-synonymous |
| 43305 | *rpoC2* | exon | 2747 | C>U | UCG>UUG | 2 | Ser>Leu | hydrophobic | Non-synonymous |
| 45135 | *rps2* | exon | 134 | C>U | ACA>AUA | 2 | Thr>Ile | hydrophobic | Non-synonymous |
| 45249 | *rps2* | exon | 248 | C>U | UCA>UUA | 2 | Ser>Leu | hydrophobic | Non-synonymous |
| 48598 | *atpF* | exon | 92 | C>U | CCA>CUA | 2 | Pro>Leu | hydrophobic | Non-synonymous |
| 55717 | *rps16* | intron | - | C>U | - | - | - | - | - |
| 56316 | *rps16* | exon | 212 | C>U | UCA>UUA | 2 | Ser>Leu | hydrophobic | Non-synonymous |
| 57521 | *accD* | exon | 617 | C>U | UCG>UUG | 2 | Ser>Leu | hydrophobic | Non-synonymous |
| 58516 | *psaI* | exon | 79 | C>U | CAU>UAU | 1 | His>Tyr | hydrophobic | Non-synonymous |
| 62511 | *psbJ* | exon | 59 | G>A | ACU>AUU | 2 | Thr>Ile | hydrophobic | Non-synonymous |
| 62825 | *psbL* | exon | 2 | G>A | ACG>AUG | 2 | Thr>Met | hydrophobic | Non-synonymous |
| 62892 | *psbF* | exon | 77 | G>A | UCU>UUU | 2 | Ser>Phe | hydrophobic | Non-synonymous |
| 62963 | *psbF* | exon | 6 | G>A | ACC>ACU | 3 | Thr>Thr | - | Synonymous |
| 62970 | *IGR* | IGR | - | G>A | - | - | - | - | - |
| 66646 | *rps18* | exon | 221 | C>U | UCG>UUG | 2 | Ser>Leu | hydrophobic | Non-synonymous |
| 68554 | *clpP* | exon | 559 | G>A | CAC>UAC | 1 | His>Tyr | hydrophobic | Non-synonymous |
| 74903 | *petB* | exon | 611 | C>U | UCA>UUA | 2 | Ser>Leu | hydrophobic | Non-synonymous |
| 77390 | *rpoA* | exon | 200 | G>A | UCA>UUA | 2 | Ser>Leu | hydrophobic | Non-synonymous |
| 85061 | *rpl23* | exon | 89 | G>A | UCA>UUA | 2 | Ser>Leu | hydrophobic | Non-synonymous |
| 89425 | *ycf2* | exon | 3671 | C>U | UCA>UUA | 2 | Ser>Leu | hydrophobic | Non-synonymous |
| 93752 | *ndhB* | exon | 1427 | G>A | CCA>CUA | 2 | Pro>Leu | hydrophobic | Non-synonymous |
| 93978 | *ndhB* | exon | 1201 | G>A | CAU>UAU | 1 | His>Tyr | hydrophobic | Non-synonymous |
| 94121 | *ndhB* | exon | 1058 | G>A | UCA>UUA | 2 | Ser>Leu | hydrophobic | Non-synonymous |
| 94397 | *ndhB* | exon | 782 | G>A | UCA>UUA | 2 | Ser>Leu | hydrophobic | Non-synonymous |
| 94403 | *ndhB* | exon | 776 | G>A | UCA>UUA | 2 | Ser>Leu | hydrophobic | Non-synonymous |
| 95178 | *ndhB* | exon | 692 | G>A | UCU>UUU | 2 | Ser>Phe | hydrophobic | Non-synonymous |
| 95187 | *ndhB* | exon | 683 | G>A | CCA>CUA | 2 | Pro>Leu | hydrophobic | Non-synonymous |
| 95338 | *ndhB* | exon | 532 | G>A | CAU>UAU | 1 | His>Tyr | hydrophobic | Non-synonymous |
| 95382 | *ndhB* | exon | 488 | G>A | ACG>AUG | 2 | Thr>Met | hydrophobic | Non-synonymous |
| 95775 | *ndhB* | exon | 95 | G>A | UCA>UUA | 2 | Ser>Leu | hydrophobic | Non-synonymous |
| 96986 | *IGR* | IGR | - | G>A | - | - | - | - | - |
| 115990 | *ndhA* | exon | 341 | C>U | UCA>UUA | 2 | Ser>Leu | hydrophobic | Non-synonymous |
| 117990 | *ndhA* | exon | 1073 | C>U | UCU>UUU | 2 | Ser>Phe | hydrophobic | Non-synonymous |
| 119881 | *ndhE* | exon | 233 | C>U | CCG>CUG | 2 | Pro>Leu | hydrophobic | Non-synonymous |
| 120626 | *ndhD* | exon | 2 | C>U | ACG>AUG | 2 | Thr>Met | hydrophobic | Non-synonymous |
| 121007 | *ndhD* | exon | 383 | C>U | ACA>AUA | 2 | Thr>Ile | hydrophobic | Non-synonymous |
| 121298 | *ndhD* | exon | 674 | C>U | UCA>UUA | 2 | Ser>Leu | hydrophobic | Non-synonymous |
| 121502 | *ndhD* | exon | 878 | C>U | UCA>UUA | 2 | Ser>Leu | hydrophobic | Non-synonymous |
| 121922 | *ndhD* | exon | 1298 | C>U | UCA>UUA | 2 | Ser>Leu | hydrophobic | Non-synonymous |
| 138424 | IGR | IGR | - | C>U | - | - | - | - | - |
| 139635 | *ndhB* | exon | 95 | C>U | UCA>UUA | 2 | Ser>Leu | hydrophobic | Non-synonymous |
| 140028 | *ndhB* | exon | 488 | C>U | ACG>AUG | 2 | Thr>Met | hydrophobic | Non-synonymous |
| 140072 | *ndhB* | exon | 532 | C>U | CAU>UAU | 1 | His>Tyr | hydrophobic | Non-synonymous |
| 140223 | *ndhB* | exon | 683 | C>U | CCA>CUA | 2 | Pro>Leu | hydrophobic | Non-synonymous |
| 140232 | *ndhB* | exon | 692 | C>U | UCU>UUU | 2 | Ser>Phe | hydrophobic | Non-synonymous |
| 141007 | *ndhB* | exon | 776 | C>U | UCA>UUA | 2 | Ser>Leu | hydrophobic | Non-synonymous |
| 141013 | *ndhB* | exon | 782 | C>U | UCA>UUA | 2 | Ser>Leu | hydrophobic | Non-synonymous |
| 141289 | *ndhB* | exon | 1058 | C>U | UCA>UUA | 2 | Ser>Leu | hydrophobic | Non-synonymous |
| 141432 | *ndhB* | exon | 1201 | C>U | CAU>UAU | 1 | His>Tyr | hydrophobic | Non-synonymous |
| 141658 | *ndhB* | exon | 1427 | C>U | CCA>CUA | 2 | Pro>Leu | hydrophobic | Non-synonymous |
| 145985 | *ycf2* | exon | 3671 | G>A | UCA>UUA | 2 | Ser>Leu | hydrophobic | Non-synonymous |
| 150349 | *rpl23* | exon | 89 | C>U | UCA>UUA | 2 | Ser>Leu | hydrophobic | Non-synonymous |

**Supplementary Table S3 Statistics of All Mitochondrial Editing Sites under Drought Stress**

| Chromosomal location | Gene name | Genotype | Position on the gene | Codon change | Amino acid changes | Codon change position | Protein changes | Hydrophobicity change |
| --- | --- | --- | --- | --- | --- | --- | --- | --- |
| 4086 | IGR | IGR | - | C>U | - | - | - | - |
| 4230 | *nad9* | exon | 14 | C>U | UCC>UUC | 2 | Ser>Phe | hydrophobic |
| 4308 | *nad9* | exon | 92 | C>U | UCU>UUU | 2 | Ser>Phe | hydrophobic |
| 4383 | *nad9* | exon | 167 | C>U | UCG>UUG | 2 | Ser>Leu | hydrophobic |
| 4514 | *nad9* | exon | 298 | C>U | CCG>UCG | 1 | Pro>Ser | hydrophobic |
| 4544 | *nad9* | exon | 328 | C>U | CGG>UGG | 1 | Arg>Trp | hydrophobic |
| 4584 | *nad9* | exon | 368 | C>U | UCC>UUC | 2 | Ser>Phe | hydrophobic |
| 4614 | *nad9* | exon | 398 | C>U | UCA>UUA | 2 | Ser>Leu | hydrophobic |
| 4655 | *nad9* | exon | 439 | C>U | CUU>UUU | 1 | Leu>Phe | Hydrophilic |
| 37005 | *nad4* | exon | 1433 | G>A | CCG>CUG | 2 | Pro>Leu | hydrophobic |
| 37021 | *nad4* | exon | 1417 | G>A | CAC>UAC | 1 | His>Tyr | hydrophobic |
| 37033 | *nad4* | exon | 1405 | G>A | CGG>UGG | 1 | Arg>Trp | hydrophobic |
| 37043 | *nad4* | Intron | - | G>A | - | - | - | - |
| 37580 | *nad4* | Intron | - | G>A | - | - | - | - |
| 39093 | *nad4* | Intron | - | G>A | - | - | - | - |
| 39634 | *nad4* | exon | 1373 | G>A | UCC>UUC | 2 | Ser>Phe | hydrophobic |
| 39652 | *nad4* | exon | 1355 | G>A | CCA>CUA | 2 | Pro>Leu | hydrophobic |
| 39796 | *nad4* | exon | 1211 | G>A | UCA>UUA | 2 | Ser>Leu | hydrophobic |
| 39801 | *nad4* | exon | 1206 | G>A | CCC>CUU | 3 | Pro>Leu | hydrophobic |
| 39802 | *nad4* | exon | 1205 | G>A | CCC>CUU | 2 | Pro>Leu | hydrophobic |
| 39835 | *nad4* | exon | 1172 | G>A | UCA>UUA | 2 | Ser>Leu | hydrophobic |
| 39859 | *nad4* | exon | 1148 | G>A | UCU>UUU | 2 | Ser>Phe | hydrophobic |
| 39865 | *nad4* | exon | 1142 | G>A | UCC>UUC | 2 | Ser>Phe | hydrophobic |
| 39878 | *nad4* | exon | 1129 | G>A | CUC>UUC | 1 | Leu>Phe | Hydrophilic |
| 39974 | *nad4* | exon | 1033 | G>A | CCU>UCU | 1 | Pro>Ser | hydrophobic |
| 39997 | *nad4* | exon | 1010 | G>A | CCG>UUG | 2 | Pro>Leu | hydrophobic |
| 39998 | *nad4* | exon | 1009 | G>A | CCG>UUG | 1 | Pro>Leu | hydrophobic |
| 40000 | *nad4* | exon | 1007 | G>A | CCA>CUA | 2 | Pro>Leu | hydrophobic |
| 42627 | *nad4* | Intron | - | G>A | - | - | - | - |
| 43402 | *nad4* | exon | 896 | G>A | UCA>UUA | 2 | Ser>Leu | hydrophobic |
| 43441 | *nad4* | exon | 857 | G>A | CCA>UUA | 2 | Pro>Leu | hydrophobic |
| 43442 | *nad4* | exon | 856 | G>A | CCA>UUA | 1 | Pro>Leu | hydrophobic |
| 43462 | *nad4* | exon | 836 | G>A | UCC>UUC | 2 | Ser>Phe | hydrophobic |
| 43514 | *nad4* | exon | 784 | G>A | CAC>UAC | 1 | His>Tyr | hydrophobic |
| 43528 | *nad4* | exon | 770 | G>A | UCA>UUA | 2 | Ser>Leu | hydrophobic |
| 43531 | *nad4* | exon | 767 | G>A | CCU>CUU | 2 | Pro>Leu | hydrophobic |
| 43639 | *nad4* | exon | 659 | G>A | UCU>UUU | 2 | Ser>Phe | hydrophobic |
| 43690 | *nad4* | exon | 608 | G>A | UCA>UUA | 2 | Ser>Leu | hydrophobic |
| 43724 | *nad4* | exon | 574 | G>A | CUU>UUU | 1 | Leu>Phe | Hydrophilic |
| 44025 | *nad4* | Intron | - | G>A | - | - | - | - |
| 45108 | *nad4* | Intron | - | G>A | - | - | - | - |
| 45270 | *nad4* | exon | 437 | G>A | CCC>UUC | 2 | Pro>Phe | hydrophobic |
| 45271 | *nad4* | exon | 436 | G>A | CCC>UUC | 1 | Pro>Phe | hydrophobic |
| 45274 | *nad4* | exon | 433 | G>A | CUU>UUU | 1 | Leu>Phe | Hydrophilic |
| 45291 | *nad4* | exon | 416 | G>A | CCU>CUU | 2 | Pro>Leu | hydrophobic |
| 45304 | *nad4* | exon | 403 | G>A | CGC>UGC | 1 | Arg>Cys | hydrophobic |
| 45331 | *nad4* | exon | 376 | G>A | CGU>UGU | 1 | Arg>Cys | hydrophobic |
| 45339 | *nad4* | exon | 368 | G>A | UCU>UUU | 2 | Ser>Phe | hydrophobic |
| 45345 | *nad4* | exon | 362 | G>A | ACA>AUA | 2 | Thr>Ile | hydrophobic |
| 45404 | *nad4* | exon | 303 | G>A | AUC>AUU | 3 | Ile>Ile | - |
| 45510 | *nad4* | exon | 197 | G>A | UCU>UUU | 2 | Ser>Phe | hydrophobic |
| 45541 | *nad4* | exon | 166 | G>A | CGG>UGG | 1 | Arg>Trp | hydrophobic |
| 45543 | *nad4* | exon | 164 | G>A | CCU>CUU | 2 | Pro>Leu | hydrophobic |
| 45549 | *nad4* | exon | 158 | G>A | CCU>CUU | 2 | Pro>Leu | hydrophobic |
| 45600 | *nad4* | exon | 107 | G>A | CCG>CUG | 2 | Pro>Leu | hydrophobic |
| 45630 | *nad4* | exon | 77 | G>A | CCU>CUU | 2 | Pro>Leu | hydrophobic |
| 45663 | *nad4* | exon | 44 | G>A | CCU>CUU | 2 | Pro>Leu | hydrophobic |
| 45678 | *nad4* | exon | 29 | G>A | UCU>UUU | 2 | Ser>Phe | hydrophobic |
| 47407 | IGR | IGR | - | G>A | - | - | - | - |
| 47878 | IGR | IGR | - | G>A | - | - | - | - |
| 65178 | IGR | IGR | - | C>U | - | - | - | - |
| 67317 | IGR | IGR | - | C>U | - | - | - | - |
| 76077 | IGR | IGR | - | C>U | - | - | - | - |
| 83543 | IGR | IGR | - | C>U | - | - | - | - |
| 83745 | *atp8* | exon | 47 | C>U | UCA>UUA | 2 | Ser>Leu | hydrophobic |
| 83756 | *atp8* | exon | 58 | C>U | CUC>UUC | 1 | Leu>Phe | Hydrophilic |
| 83774 | *atp8* | exon | 76 | C>U | CCC>UUC | 1 | Pro>Phe | hydrophobic |
| 83775 | *atp8* | exon | 77 | C>U | CCC>UUC | 2 | Pro>Phe | hydrophobic |
| 84118 | *atp8* | exon | 420 | C>U | AUC>AUU | 3 | Ile>Ile | - |
| 84133 | *atp8* | exon | 435 | C>U | UAC>UAU | 3 | Tyr>Tyr | - |
| 84153 | *atp8* | exon | 455 | C>U | CCA>CUA | 2 | Pro>Leu | hydrophobic |
| 97223 | *atp6*-1 | exon | 193 | C>U | CCC>UCU | 1 | Pro>Ser | hydrophobic |
| 97225 | *atp6*-1 | exon | 195 | C>U | CCC>UCU | 3 | Pro>Ser | hydrophobic |
| 97353 | *atp6*-1 | exon | 323 | C>U | CCG>CUG | 2 | Pro>Leu | hydrophobic |
| 97413 | *atp6*-1 | exon | 383 | C>U | UCG>UUG | 2 | Ser>Leu | hydrophobic |
| 97431 | *atp6*-1 | exon | 401 | C>U | UCG>UUG | 2 | Ser>Leu | hydrophobic |
| 97446 | *atp6*-1 | exon | 416 | C>U | CCC>CUU | 2 | Pro>Leu | hydrophobic |
| 97447 | *atp6*-1 | exon | 417 | C>U | CCC>CUU | 2 | Pro>Leu | hydrophobic |
| 97578 | *atp6*-1 | exon | 548 | C>U | UCA>UUA | 2 | Ser>Leu | hydrophobic |
| 97637 | *atp6*-1 | exon | 607 | C>U | CCU>UCU | 1 | Pro>Ser | hydrophobic |
| 97640 | *atp6*-1 | exon | 610 | C>U | CAU>UAU | 1 | His>Tyr | hydrophobic |
| 97662 | *atp6*-1 | exon | 632 | C>U | UCA>UUA | 2 | Ser>Leu | hydrophobic |
| 97704 | *atp6*-1 | exon | 674 | C>U | UCA>UUA | 2 | Ser>Leu | hydrophobic |
| 97725 | *atp6*-1 | exon | 695 | C>U | UCC>UUC | 2 | Ser>Phe | hydrophobic |
| 97812 | *atp6*-1 | exon | 782 | C>U | CCG>CUG | 2 | Pro>Leu | hydrophobic |
| 97833 | *atp6*-1 | exon | 803 | C>U | UCA>UUA | 2 | Ser>Leu | hydrophobic |
| 97841 | *atp6*-1 | exon | 811 | C>U | CAU>UAU | 2 | His>Tyr | hydrophobic |
| 97848 | *atp6*-1 | exon | 818 | C>U | UCU>UUU | 2 | Ser>Phe | hydrophobic |
| 97895 | *atp6*-1 | exon | 865 | C>U | CAA>UAA | 1 | Gln>* | - |
| 104607 | IGR | IGR | - | G>A | - | - | - | - |
| 104751 | IGR | IGR | - | G>U | - | - | - | - |
| 104761 | IGR | IGR | - | U>G | - | - | - | - |
| 104772 | IGR | IGR | - | U>C | - | - | - | - |
| 104777 | IGR | IGR | - | C>U | - | - | - | - |
| 106200 | IGR | IGR | - | G>A | - | - | - | - |
| 119741 | *rpl16* | exon | 512 | G>A | UCG>UUG | 2 | Ser>Leu | hydrophobic |
| 119747 | *rpl16* | exon | 506 | G>A | CCA>CUA | 2 | Pro>Leu | hydrophobic |
| 119827 | *rpl16* | exon | 426 | G>A | UCC>UCU | 3 | Ser>Ser | - |
| 120044 | *rpl16* | exon | 209 | G>A | ACU>AUU | 2 | Thr>Ile | hydrophobic |
| 120192 | *rpl16* | exon | 61 | G>A | CAG>UAG | 1 | Gln>* | - |
| 120256 | *rps3* | exon | 1552 | G>A | CGU>UGU | 1 | Arg>Cys | hydrophobic |
| 120309 | *rps3* | exon | 1499 | G>A | UCA>UUA | 2 | Ser>Leu | hydrophobic |
| 120447 | *rps3* | exon | 1361 | G>A | CCG>CUG | 2 | Pro>Leu | hydrophobic |
| 120780 | *rps3* | exon | 1028 | G>A | CCA>CUA | 2 | Pro>Leu | hydrophobic |
| 120921 | *rps3* | exon | 887 | G>A | UCG>UUG | 2 | Ser>Leu | hydrophobic |
| 121095 | *rps3* | exon | 713 | G>A | UCG>UUG | 2 | Ser>Leu | hydrophobic |
| 121296 | *rps3* | exon | 512 | G>A | UCA>UUA | 2 | Ser>Leu | hydrophobic |
| 121490 | *rps3* | exon | 318 | G>A | AUC>AUU | 3 | Ile>Ile | - |
| 123120 | *rps3* | exon | 64 | G>A | CGG>UGG | 1 | Arg>Trp | hydrophobic |
| 123263 | IGR | IGR | - | G>A | - | - | - | - |
| 123324 | IGR | IGR | - | G>A | - | - | - | - |
| 129876 | *nad2* | exon | 1457 | G>A | UCA>UUA | 2 | Ser>Leu | hydrophobic |
| 129924 | *nad2* | exon | 1409 | G>A | CCA>UUA | 2 | Pro>Leu | hydrophobic |
| 129925 | *nad2* | exon | 1408 | G>A | CCA>UUA | 1 | Pro>Leu | hydrophobic |
| 129930 | *nad2* | exon | 1403 | G>A | UCC>UUC | 2 | Ser>Phe | hydrophobic |
| 129933 | *nad2* | exon | 1400 | G>A | UCA>UUA | 2 | Ser>Leu | hydrophobic |
| 130035 | *nad2* | exon | 1298 | G>A | GCG>GUG | 2 | Ala>Val | hydrophobic |
| 131385 | *nad2* | Intron | - | G>A | - | - | - | - |
| 131403 | *nad2* | Intron | - | G>A | - | - | - | - |
| 131564 | *nad2* | exon | 1246 | G>A | CCA>UCA | 1 | Pro>Ser | hydrophobic |
| 131683 | *nad2* | exon | 1127 | G>A | UCG>UUG | 2 | Ser>Leu | hydrophobic |
| 131848 | *nad2* | exon | 962 | G>A | ACU>AUU | 2 | Thr>Ile | hydrophobic |
| 131852 | *nad2* | exon | 958 | G>A | CGU>UGU | 1 | Arg>Cys | hydrophobic |
| 131882 | *nad2* | exon | 928 | G>A | CAU>UAU | 1 | His>Tyr | hydrophobic |
| 131890 | *nad2* | exon | 920 | G>A | CCU>CUU | 2 | Pro>Leu | hydrophobic |
| 132001 | *nad2* | exon | 809 | G>A | UCU>UUU | 2 | Ser>Phe | hydrophobic |
| 132010 | *nad2* | exon | 800 | G>A | UCA>UUA | 2 | Ser>Leu | hydrophobic |
| 132152 | *nad2* | Intron | - | G>A | - | - | - | - |
| 132158 | *nad2* | Intron | - | G>A | - | - | - | - |
| 132299 | *nad2* | Intron | - | G>A | - | - | - | - |
| 132692 | *IGR* | IGR | - | G>A | - | - | - | - |
| 133686 | *nad2* | Intron | - | G>A | - | - | - | - |
| 134705 | *nad2* | exon | 677 | G>A | UCC>UUC | 2 | Ser>Phe | hydrophobic |
| 134720 | *nad2* | exon | 662 | G>A | UCU>UUU | 2 | Ser>Phe | hydrophobic |
| 134773 | *nad2* | exon | 609 | G>A | ACC>ACU | 3 | Thr>Thr | - |
| 134951 | IGR | IGR | - | G>A | - | - | - | - |
| 140275 | *atp9* | exon | 20 | C>U | UCA>UUA | 2 | Ser>Leu | hydrophobic |
| 140305 | *atp9* | exon | 50 | C>U | UCA>UUA | 2 | Ser>Leu | hydrophobic |
| 148911 | *nad1* | exon | 953 | G>A | UCA>UUA | 2 | Ser>Leu | hydrophobic |
| 148936 | *nad1* | exon | 928 | G>A | CGG>UGG | 1 | Arg>Trp | hydrophobic |
| 148966 | *nad1* | exon | 898 | G>A | CGG>UGG | 1 | Arg>Trp | hydrophobic |
| 149109 | *nad1* | exon | 755 | G>A | CCG>CUG | 2 | Pro>Leu | hydrophobic |
| 149121 | *nad1* | exon | 743 | G>A | CCA>CUA | 2 | Pro>Leu | hydrophobic |
| 149124 | *nad1* | exon | 740 | G>A | UCU>UUU | 2 | Ser>Phe | hydrophobic |
| 149130 | *nad1* | exon | 734 | G>A | UCG>UUG | 2 | Ser>Leu | hydrophobic |
| 149139 | *nad1* | exon | 725 | G>A | CCA>CUA | 2 | Pro>Leu | hydrophobic |
| 149153 | IGR | IGR | - | G>A | - | - | - | - |
| 149632 | IGR | IGR | - | G>A | - | - | - | - |
| 149793 | *matR* | exon | 1851 | G>A | GUC>GUU | 3 | Val>Val | - |
| 149803 | *matR* | exon | 1841 | G>A | UCA>UUA | 2 | Ser>Leu | hydrophobic |
| 149821 | *matR* | exon | 1823 | G>A | CCC>CUC | 2 | Pro>Leu | hydrophobic |
| 149891 | *matR* | exon | 1753 | G>A | CAC>UAC | 1 | His>Tyr | hydrophobic |
| 149913 | *matR* | exon | 1731 | G>A | UAC>UAU | 3 | Tyr>Tyr | - |
| 149927 | *matR* | exon | 1717 | G>A | CGC>UGC | 1 | Arg>Cys | hydrophobic |
| 149947 | *matR* | exon | 1697 | G>A | CCU>CUU | 2 | Pro>Leu | hydrophobic |
| 149968 | *matR* | exon | 1676 | G>A | UCU>UUU | 2 | Ser>Phe | hydrophobic |
| 150102 | *matR* | exon | 1542 | G>A | CCC>CCU | 3 | Pro>Pro | - |
| 150583 | *matR* | exon | 1061 | G>A | CCC>CUC | 2 | Pro>Leu | hydrophobic |
| 150733 | *matR* | exon | 911 | G>A | GCC>GUC | 2 | Ala>Val | hydrophobic |
| 150892 | *matR* | exon | 752 | G>A | GCC>GUC | 2 | Ala>Val | hydrophobic |
| 151240 | *matR* | exon | 404 | G>A | UCG>UUG | 2 | Ser>Leu | hydrophobic |
| 151318 | *matR* | exon | 326 | G>A | CCA>CUA | 2 | Pro>Leu | hydrophobic |
| 151408 | *matR* | exon | 236 | G>A | UCC>UUC | 2 | Ser>Phe | hydrophobic |
| 151451 | *matR* | exon | 193 | G>A | CCA>UCA | 1 | Pro>Ser | hydrophobic |
| 151497 | *matR* | exon | 147 | G>A | UUC>UUU | 3 | Phe>Phe | - |
| 151612 | *matR* | exon | 32 | G>A | UCC>UUC | 2 | Ser>Phe | hydrophobic |
| 151687 | IGR | IGR | - | G>A | - | - | - | - |
| 152349 | *nad1* | exon | 55 | G>A | CUU>UUU | 2 | Leu>Phe | hydrophobic |
| 152360 | IGR | IGR | - | G>A | - | - | - | - |
| 158540 | IGR | IGR | - | C>U | - | - | - | - |
| 161260 | IGR | IGR | - | C>U | - | - | - | - |
| 162009 | IGR | IGR | - | U>C | - | - | - | - |
| 162057 | IGR | IGR | - | A>U | - | - | - | - |
| 164405 | *cox2* | exon | 376 | G>A | CGG>UGG | 1 | Arg>Trp | hydrophobic |
| 164531 | *cox2* | exon | 250 | G>A | CGG>UGG | 1 | Arg>Trp | hydrophobic |
| 164621 | *cox2* | exon | 160 | G>A | CGG>UGG | 1 | Arg>Trp | hydrophobic |
| 166991 | IGR | IGR | - | G>A | - | - | - | - |
| 173635 | IGR | IGR | - | C>U | - | - | - | - |
| 181462 | IGR | IGR | - | C>U | - | - | - | - |
| 181647 | *atp8* | exon | 30 | C>U | UUC>UUU | 3 | Phe>Phe | - |
| 181664 | *atp8* | exon | 47 | C>U | UCA>UUA | 2 | Ser>Leu | hydrophobic |
| 181675 | *atp8* | exon | 58 | C>U | CUC>UUC | 1 | Leu>Phe | Hydrophilic |
| 181693 | *atp8* | exon | 76 | C>U | CCC>UUC | 1 | Pro>Phe | hydrophobic |
| 181694 | *atp8* | exon | 77 | C>U | CCC>UUC | 2 | Pro>Phe | hydrophobic |
| 182037 | *atp8* | exon | 420 | C>U | AUC>AUU | 3 | Ile>Ile | - |
| 182052 | *atp8* | exon | 435 | C>U | UAC>UAU | 3 | Tyr>Tyr | - |
| 182072 | *atp8* | exon | 455 | C>U | CCA>CUA | 2 | Pro>Leu | hydrophobic |
| 185370 | IGR | IGR | - | C>U | - | - | - | - |
| 195142 | *atp6*-1 | exon | 193 | C>U | CCC>UCC | 1 | Pro>Ser | hydrophobic |
| 195144 | *atp6*-1 | exon | 195 | C>U | CCC>UCU | 3 | Pro>Ser | hydrophobic |
| 195272 | *atp6*-1 | exon | 323 | C>U | CCG>CUG | 2 | Pro>Leu | hydrophobic |
| 195332 | *atp6*-1 | exon | 383 | C>U | UCG>UUG | 2 | Ser>Leu | hydrophobic |
| 195350 | *atp6*-1 | exon | 401 | C>U | UCG>UUG | 2 | Ser>Leu | hydrophobic |
| 195365 | *atp6*-1 | exon | 416 | C>U | CCC>CUU | 2 | Pro>Leu | hydrophobic |
| 195366 | *atp6*-1 | exon | 417 | C>U | CCC>CUU | 2 | Pro>Leu | hydrophobic |
| 195497 | *atp6*-1 | exon | 548 | C>U | UCA>UUA | 2 | Ser>Leu | hydrophobic |
| 195556 | *atp6*-1 | exon | 607 | C>U | CCU>UCU | 1 | Pro>Ser | hydrophobic |
| 195559 | *atp6*-1 | exon | 610 | C>U | CAU>UAU | 1 | His>Tyr | hydrophobic |
| 195581 | *atp6*-1 | exon | 632 | C>U | UCA>UUA | 2 | Ser>Leu | hydrophobic |
| 195623 | *atp6*-1 | exon | 674 | C>U | UCA>UUA | 2 | Ser>Leu | hydrophobic |
| 195644 | *atp6*-1 | exon | 695 | C>U | UCC>UUC | 2 | Ser>Phe | hydrophobic |
| 195731 | *atp6*-1 | exon | 782 | C>U | CCG>CUG | 2 | Pro>Leu | hydrophobic |
| 195752 | *atp6*-1 | exon | 803 | C>U | UCA>UUA | 2 | Ser>Leu | hydrophobic |
| 195760 | *atp6*-1 | exon | 811 | C>U | CAU>UAU | 1 | His>Tyr | hydrophobic |
| 195814 | *atp6*-1 | exon | 865 | C>U | CAA>UAA | 1 | Gln>* | - |
| 202526 | IGR | IGR | - | G>A | - | - | - | - |
| 202589 | IGR | IGR | - | A>C | - | - | - | - |
| 202670 | IGR | IGR | - | G>U | - | - | - | - |
| 202680 | IGR | IGR | - | U>G | - | - | - | - |
| 202691 | IGR | IGR | - | U>C | - | - | - | - |
| 204119 | IGR | IGR | - | G>A | - | - | - | - |
| 205435 | *atp1* | exon | 1029 | G>A | ACC>ACU | 3 | Thr>Thr | - |
| 206218 | *atp1* | exon | 246 | G>A | ACC>ACU | 3 | Thr>Thr | - |
| 206570 | IGR | IGR | - | G>A | - | - | - | - |
| 206612 | IGR | IGR | - | G>A | - | - | - | - |
| 206633 | IGR | IGR | - | G>A | - | - | - | - |
| 206648 | IGR | IGR | - | G>A | - | - | - | - |
| 206657 | IGR | IGR | - | G>A | - | - | - | - |
| 206679 | IGR | IGR | - | G>A | - | - | - | - |
| 206763 | IGR | IGR | - | G>A | - | - | - | - |
| 206773 | IGR | IGR | - | G>A | - | - | - | - |
| 208197 | IGR | IGR | - | G>A | - | - | - | - |
| 208385 | IGR | IGR | - | G>A | - | - | - | - |
| 208791 | *nad5* | exon | 1310 | G>A | UCA>UUA | 2 | Ser>Leu | hydrophobic |
| 209226 | *nad5* | exon | 875 | G>A | ACG>AUG | 2 | Thr>Met | hydrophobic |
| 209238 | *nad5* | exon | 863 | G>A | UCU>UUU | 2 | Ser>Phe | hydrophobic |
| 209266 | *nad5* | exon | 835 | G>A | CCA>UCA | 1 | Pro>Ser | hydrophobic |
| 209376 | *nad5* | exon | 725 | G>A | UCA>UUA | 2 | Ser>Leu | hydrophobic |
| 209388 | *nad5* | exon | 713 | G>A | UCG>UUG | 2 | Ser>Leu | hydrophobic |
| 209425 | *nad5* | exon | 676 | G>A | CUU>UUU | 1 | Leu>Phe | Hydrophilic |
| 209472 | *nad5* | exon | 629 | G>A | UCU>UUU | 2 | Ser>Phe | hydrophobic |
| 209493 | *nad5* | exon | 608 | G>A | GCC>GUC | 2 | Ala>Val | hydrophobic |
| 209503 | *nad5* | exon | 598 | G>A | CGU>UGU | 1 | Arg>Cys | hydrophobic |
| 209548 | *nad5* | exon | 553 | G>A | CGU>UGU | 1 | Arg>Cys | hydrophobic |
| 209553 | *nad5* | exon | 548 | G>A | UCG>UUG | 2 | Ser>Leu | hydrophobic |
| 209562 | *nad5* | exon | 539 | G>A | CCU>CUU | 2 | Pro>Leu | hydrophobic |
| 209595 | *nad5* | exon | 506 | G>A | CCU>CUU | 2 | Pro>Leu | hydrophobic |
| 209607 | *nad5* | exon | 494 | G>A | ACA>AUA | 2 | Thr>Ile | hydrophobic |
| 209642 | *nad5* | exon | 459 | G>A | UUC>UUU | 3 | Phe>Phe | - |
| 209703 | *nad5* | exon | 398 | G>A | UCU>UUU | 2 | Ser>Phe | hydrophobic |
| 209727 | *nad5* | exon | 374 | G>A | CCA>CUA | 2 | Pro>Leu | hydrophobic |
| 209743 | *nad5* | exon | 358 | G>A | CUU>UUU | 1 | Leu>Phe | Hydrophilic |
| 209829 | *nad5* | exon | 272 | G>A | UCC>UUC | 2 | Ser>Phe | hydrophobic |
| 209859 | *nad5* | exon | 242 | G>A | CCG>CUG | 2 | Pro>Leu | hydrophobic |
| 209880 | *nad5* | Intron | - | G>A | - | - | - | - |
| 209910 | *nad5* | Intron | - | G>A | - | - | - | - |
| 210805 | *nad5* | exon | 155 | G>A | CCG>CUG | 2 | Pro>Leu | hydrophobic |
| 211087 | IGR | IGR | - | G>A | - | - | - | - |
| 211567 | *rps1* | exon | 212 | G>A | UCC>UUC | 2 | Ser>Phe | hydrophobic |
| 211618 | *rps1* | exon | 161 | G>A | CCU>CUU | 2 | Pro>Leu | hydrophobic |
| 211734 | *rps1* | exon | 45 | G>A | UCC>UCU | 3 | Ser>Ser | - |
| 211845 | IGR | IGR | - | G>A | - | - | - | - |
| 212317 | IGR | IGR | - | G>A | - | - | - | - |
| 212549 | *rps4* | exon | 1009 | G>A | CGG>UGG | 1 | Arg>Trp | hydrophobic |
| 212554 | *rps4* | exon | 1004 | G>A | CCU>CUU | 2 | Pro>Leu | hydrophobic |
| 212563 | *rps4* | exon | 995 | G>A | CCA>CUA | 2 | Pro>Leu | hydrophobic |
| 212629 | *rps4* | exon | 929 | G>A | CCA>CUA | 2 | Pro>Leu | hydrophobic |
| 212639 | *rps4* | exon | 919 | G>A | CAU>UAU | 1 | His>Tyr | hydrophobic |
| 212650 | *rps4* | exon | 908 | G>A | UCG>UUG | 2 | Ser>Leu | hydrophobic |
| 212724 | *rps4* | exon | 834 | G>A | ACC>ACU | 3 | Thr>Thr | - |
| 213085 | *rps4* | exon | 473 | G>A | UCA>UUA | 2 | Ser>Leu | hydrophobic |
| 213103 | *rps4* | exon | 455 | G>A | UCU>UUU | 2 | Ser>Phe | hydrophobic |
| 213232 | *rps4* | exon | 326 | G>A | CCG>CUG | 2 | Pro>Leu | hydrophobic |
| 213260 | *rps4* | exon | 298 | G>A | CGU>UGU | 1 | Arg>Cys | hydrophobic |
| 213277 | *rps4* | exon | 281 | G>A | CCG>CUG | 2 | Pro>Leu | hydrophobic |
| 213289 | *rps4* | exon | 269 | G>A | UCG>UUG | 2 | Ser>Leu | hydrophobic |
| 213301 | *rps4* | exon | 257 | G>A | CCA>CUA | 2 | Pro>Leu | hydrophobic |
| 213374 | *rps4* | exon | 184 | G>A | CAU>UAU | 1 | His>Tyr | hydrophobic |
| 213383 | *rps4* | exon | 175 | G>A | CCC>UCC | 1 | Pro>Ser | hydrophobic |
| 213403 | *rps4* | exon | 155 | G>A | UCA>UUA | 2 | Ser>Leu | hydrophobic |
| 213434 | *rps4* | exon | 124 | G>A | CCG>UCG | 1 | Pro>Ser | hydrophobic |
| 213518 | *rps4* | exon | 40 | G>A | CGG>UGG | 1 | Arg>Trp | hydrophobic |
| 213529 | *rps4* | exon | 29 | G>A | UCA>UUA | 2 | Ser>Leu | hydrophobic |
| 213582 | IGR | IGR | - | G>A | - | - | - | - |
| 213820 | IGR | IGR | - | G>A | - | - | - | - |
| 216130 | IGR | IGR | - | C>U | - | - | - | - |
| 216838 | *ccmFn* | exon | 41 | C>U | CCG>CUG | 2 | Pro>Leu | hydrophobic |
| 216898 | *ccmFn* | exon | 101 | C>U | CCU>CUU | 2 | Pro>Leu | hydrophobic |
| 216937 | *ccmFn* | exon | 140 | C>U | UCG>UUG | 2 | Ser>Leu | hydrophobic |
| 216942 | *ccmFn* | exon | 145 | C>U | CGU>UGU | 1 | Arg>Cys | hydrophobic |
| 216951 | *ccmFn* | exon | 154 | C>U | CCU>UCU | 1 | Pro>Ser | hydrophobic |
| 217048 | *ccmFn* | exon | 251 | C>U | UCA>UUA | 2 | Ser>Leu | hydrophobic |
| 217056 | *ccmFn* | exon | 259 | C>U | CGG>UGG | 1 | Arg>Trp | hydrophobic |
| 217063 | *ccmFn* | exon | 266 | C>U | CCA>CUA | 2 | Pro>Leu | hydrophobic |
| 217065 | *ccmFn* | exon | 268 | C>U | CGU>UGU | 1 | Arg>Cys | hydrophobic |
| 217079 | *ccmFn* | exon | 282 | C>U | UUC>UUU | 3 | Phe>Phe | - |
| 217083 | *ccmFn* | exon | 286 | C>U | CUU>UUU | 1 | Leu>Phe | Hydrophilic |
| 217162 | *ccmFn* | exon | 365 | C>U | UCG>UUG | 2 | Ser>Leu | hydrophobic |
| 217169 | *ccmFn* | exon | 372 | C>U | UUC>UUU | 3 | Phe>Phe | - |
| 217310 | *ccmFn* | exon | 513 | C>U | GCC>GCU | 3 | Ala>Ala | - |
| 217356 | *ccmFn* | exon | 559 | C>U | CCU>UCU | 1 | Pro>Ser | hydrophobic |
| 217507 | *ccmFn* | exon | 710 | C>U | CCU>CUU | 2 | Pro>Leu | hydrophobic |
| 217516 | *ccmFn* | exon | 719 | C>U | UCA>UUA | 2 | Ser>Leu | hydrophobic |
| 217554 | *ccmFn* | exon | 757 | C>U | CGU>UGU | 1 | Arg>Cys | hydrophobic |
| 217576 | *ccmFn* | exon | 779 | C>U | UCA>UUA | 2 | Ser>Leu | hydrophobic |
| 217588 | *ccmFn* | exon | 791 | C>U | CCA>CUA | 2 | Pro>Leu | hydrophobic |
| 217603 | *ccmFn* | exon | 806 | C>U | UCA>UUA | 2 | Ser>Leu | hydrophobic |
| 217752 | *ccmFn* | exon | 955 | C>U | CGC>UGC | 1 | Arg>Cys | hydrophobic |
| 218070 | *ccmFn* | exon | 1273 | C>U | CGG>UGG | 1 | Arg>Trp | hydrophobic |
| 218098 | *ccmFn* | exon | 1301 | C>U | CCA>CUA | 2 | Pro>Leu | hydrophobic |
| 218115 | *ccmFn* | exon | 1318 | C>U | CAU>UAU | 1 | His>Tyr | hydrophobic |
| 218130 | *ccmFn* | exon | 1333 | C>U | CGG>UGG | 1 | Arg>Trp | hydrophobic |
| 218148 | *ccmFn* | exon | 1351 | C>U | CGG>UGG | 1 | Arg>Trp | hydrophobic |
| 218199 | *ccmFn* | exon | 1402 | C>U | CGU>UGU | 1 | Arg>Cys | hydrophobic |
| 218242 | *ccmFn* | exon | 1445 | C>U | UCG>UUG | 2 | Ser>Leu | hydrophobic |
| 218262 | *ccmFn* | exon | 1465 | C>U | CUU>UUU | 1 | Leu>Phe | Hydrophilic |
| 218266 | *ccmFn* | exon | 1469 | C>U | CCA>CUA | 2 | Pro>Leu | hydrophobic |
| 218278 | *ccmFn* | exon | 1481 | C>U | UCA>UUA | 2 | Ser>Leu | hydrophobic |
| 228948 | IGR | IGR | - | C>U | - | - | - | - |
| 229283 | IGR | IGR | - | C>U | - | - | - | - |
| 229287 | IGR | IGR | - | C>U | - | - | - | - |
| 229288 | IGR | IGR | - | C>U | - | - | - | - |
| 229326 | *mttB* | exon | 34 | C>U | CGG>UGG | 1 | Arg>Trp | hydrophobic |
| 229362 | *mttB* | exon | 70 | C>U | CGU>UGU | 1 | Arg>Cys | hydrophobic |
| 229374 | *mttB* | exon | 82 | C>U | CCG>UCG | 1 | Pro>Ser | hydrophobic |
| 229390 | *mttB* | exon | 98 | C>U | UCU>UUU | 2 | Ser>Phe | hydrophobic |
| 229450 | *mttB* | exon | 158 | C>U | UCA>UUA | 2 | Ser>Leu | hydrophobic |
| 229462 | *mttB* | exon | 170 | C>U | UCC>UUC | 2 | Ser>Phe | hydrophobic |
| 229464 | *mttB* | exon | 172 | C>U | CCG>UCG | 1 | Pro>Ser | hydrophobic |
| 229524 | *mttB* | exon | 232 | C>U | CAU>UAU | 1 | His>Tyr | hydrophobic |
| 229590 | *mttB* | exon | 298 | C>U | CUC>UUC | 1 | Leu>Phe | Hydrophilic |
| 229608 | *mttB* | exon | 316 | C>U | CGC>UGC | 1 | Arg>Cys | hydrophobic |
| 229638 | *mttB* | exon | 346 | C>U | CCC>UCC | 1 | Pro>Ser | hydrophobic |
| 229699 | *mttB* | exon | 407 | C>U | UCG>UUG | 2 | Ser>Leu | hydrophobic |
| 229734 | *mttB* | exon | 442 | C>U | CAU>UAU | 1 | His>Tyr | hydrophobic |
| 229759 | *mttB* | exon | 467 | C>U | UCG>UUG | 2 | Ser>Leu | hydrophobic |
| 229767 | *mttB* | exon | 475 | C>U | CCA>UCA | 1 | Pro>Ser | hydrophobic |
| 229816 | *mttB* | exon | 524 | C>U | CCA>CUA | 2 | Pro>Leu | hydrophobic |
| 229872 | *mttB* | exon | 580 | C>U | CCG>UCG | 1 | Pro>Ser | hydrophobic |
| 229922 | *mttB* | exon | 630 | C>U | AUC>AUU | 3 | Ile>Ile | - |
| 229929 | *mttB* | exon | 637 | C>U | CGU>UGU | 1 | Arg>Cys | hydrophobic |
| 229945 | *mttB* | exon | 653 | C>U | UCG>UUG | 2 | Ser>Leu | hydrophobic |
| 229975 | *mttB* | exon | 683 | C>U | UCG>UUG | 2 | Ser>Leu | hydrophobic |
| 230294 | IGR | IGR | - | C>U | - | - | - | - |
| 230699 | IGR | IGR | - | C>U | - | - | - | - |
| 230847 | IGR | IGR | - | C>U | - | - | - | - |
| 232074 | IGR | IGR | - | C>U | - | - | - | - |
| 232509 | *ccmC* | exon | 76 | C>U | CGG>UGG | 1 | Arg>Trp | hydrophobic |
| 232536 | *ccmC* | exon | 103 | C>U | CAU>UAU | 1 | His>Tyr | hydrophobic |
| 232548 | *ccmC* | exon | 115 | C>U | CGG>UGG | 1 | Arg>Trp | hydrophobic |
| 232566 | *ccmC* | exon | 133 | C>U | CUU>UUU | 1 | Leu>Phe | Hydrophilic |
| 232612 | *ccmC* | exon | 179 | C>U | GCG>GUG | 2 | Ala>Val | hydrophobic |
| 232617 | *ccmC* | exon | 184 | C>U | CGG>UGG | 1 | Arg>Trp | hydrophobic |
| 232714 | *ccmC* | exon | 281 | C>U | ACA>AUA | 2 | Thr>Ile | hydrophobic |
| 232764 | *ccmC* | exon | 331 | C>U | CGG>UGG | 1 | Arg>Trp | hydrophobic |
| 232828 | *ccmC* | exon | 395 | C>U | UCG>UUG | 2 | Ser>Leu | hydrophobic |
| 232854 | *ccmC* | exon | 421 | C>U | CGU>UGU | 1 | Arg>Cys | hydrophobic |
| 232869 | *ccmC* | exon | 436 | C>U | CCU>UCU | 1 | Pro>Ser | hydrophobic |
| 232879 | *ccmC* | exon | 446 | C>U | CCG>CUG | 2 | Pro>Leu | hydrophobic |
| 232930 | *ccmC* | exon | 497 | C>U | UCU>UUU | 2 | Ser>Phe | hydrophobic |
| 232981 | *ccmC* | exon | 548 | C>U | UCU>UUU | 2 | Ser>Phe | hydrophobic |
| 233001 | *ccmC* | exon | 568 | C>U | CCU>UCU | 1 | Pro>Ser | hydrophobic |
| 233041 | *ccmC* | exon | 608 | C>U | CCC>CUC | 2 | Pro>Leu | hydrophobic |
| 233047 | *ccmC* | exon | 614 | C>U | UCA>UUA | 2 | Ser>Leu | hydrophobic |
| 233083 | *ccmC* | exon | 650 | C>U | CCU>CUU | 2 | Pro>Leu | hydrophobic |
| 233098 | *ccmC* | exon | 665 | C>U | CCC>CUC | 2 | Pro>Leu | hydrophobic |
| 233106 | *ccmC* | exon | 673 | C>U | CCU>UCU | 1 | Pro>Ser | hydrophobic |
| 235000 | *trnP-UGG* | tRNA | 6 | C>U |  | 2 |  |  |
| 240692 | IGR | IGR | - | G>A | - | - | - | - |
| 243802 | IGR | IGR | - | G>A | - | - | - | - |
| 243858 | *nad2* | exon | 497 | G>A | UCG>UUG | 2 | Ser>Leu | hydrophobic |
| 243927 | *nad2* | exon | 428 | G>A | CCU>CUU | 2 | Pro>Leu | hydrophobic |
| 243954 | *nad2* | exon | 401 | G>A | UCA>UUA | 2 | Ser>Leu | hydrophobic |
| 243961 | *nad2* | exon | 394 | G>A | CAU>UAU | 1 | His>Tyr | hydrophobic |
| 243994 | *nad2* | exon | 361 | G>A | CCU>UCU | 1 | Pro>Ser | hydrophobic |
| 243999 | *nad2* | exon | 356 | G>A | CCA>CUA | 2 | Pro>Leu | hydrophobic |
| 244044 | *nad2* | exon | 311 | G>A | UCC>UUC | 2 | Ser>Phe | hydrophobic |
| 244047 | *nad2* | exon | 308 | G>A | UCU>UUU | 2 | Ser>Phe | hydrophobic |
| 244103 | *nad2* | exon | 252 | G>A | UUC>UUU | 3 | Phe>Phe | - |
| 244132 | *nad2* | exon | 223 | G>A | CUU>UUU | 1 | Leu>Phe | Hydrophilic |
| 244141 | *nad2* | exon | 214 | G>A | CGG>UGG | 1 | Arg>Trp | hydrophobic |
| 244262 | *nad2* | Intron | - | G>A | - | - | - | - |
| 245450 | *nad2* | exon | 2 | G>A | GCG>GUG | 2 | Ala>Val | hydrophobic |
| 245544 | *nad2* | exon | 56 | G>A | UCC>UUC | 2 | Ser>Phe | hydrophobic |
| 245574 | *nad2* | exon | 26 | G>A | UCC>UUC | 2 | Ser>Phe | hydrophobic |
| 245650 | IGR | IGR | - | G>A | - | - | - | - |
| 246346 | IGR | IGR | - | G>A | - | - | - | - |
| 246455 | IGR | IGR | - | G>A | - | - | - | - |
| 246652 | IGR | IGR | - | G>A | - | - | - | - |
| 256114 | IGR | IGR | - | C>U | - | - | - | - |
| 256408 | *nad4L* | exon | 2 | C>U | ACG>AUG | 2 | Thr>Met | hydrophobic |
| 256414 | *nad4L* | exon | 8 | C>U | CCU>CUU | 2 | Pro>Leu | hydrophobic |
| 256447 | *nad4L* | exon | 41 | C>U | UCU>UUU | 2 | Ser>Phe | hydrophobic |
| 256461 | *nad4L* | exon | 55 | C>U | CGG>UGG | 1 | Arg>Trp | hydrophobic |
| 256492 | *nad4L* | exon | 86 | C>U | CCU>CUU | 2 | Pro>Leu | hydrophobic |
| 256537 | *nad4L* | exon | 131 | C>U | UCG>UUG | 2 | Ser>Leu | hydrophobic |
| 256564 | *nad4L* | exon | 158 | C>U | UCG>UUG | 2 | Ser>Leu | hydrophobic |
| 256585 | *nad4L* | exon | 179 | C>U | UCA>UUA | 2 | Ser>Leu | hydrophobic |
| 256594 | *nad4L* | exon | 188 | C>U | UCA>UUA | 2 | Ser>Leu | hydrophobic |
| 256603 | *nad4L* | exon | 197 | C>U | UCA>UUA | 2 | Ser>Leu | hydrophobic |
| 256932 | IGR | IGR | - | C>U | - | - | - | - |
| 256935 | IGR | IGR | - | C>U | - | - | - | - |
| 275858 | *rrn26* | exon | 2952 | C>U | UCA>UUA | 2 | Ser>Leu | hydrophobic |
| 275902 | *rrn26* | exon | 2996 | C>U | CGU>UGU | 2 | Arg>Cys | hydrophobic |
| 282091 | IGR | IGR | - | G>A | - | - | - | - |
| 282154 | IGR | IGR | - | A>C | - | - | - | - |
| 282235 | IGR | IGR | - | G>U | - | - | - | - |
| 282245 | IGR | IGR | - | U>G | - | - | - | - |
| 282256 | IGR | IGR | - | U>C | - | - | - | - |
| 294701 | IGR | IGR | - | G>A | - | - | - | - |
| 298086 | IGR | IGR | - | C>U | - | - | - | - |
| 298740 | IGR | IGR | - | C>U | - | - | - | - |
| 323955 | IGR | IGR | - | G>A | - | - | - | - |
| 329472 | IGR | IGR | - | C>U | - | - | - | - |
| 330607 | *atp6*-2 | exon | 20 | C>U | UCA>UUA | 2 | Ser>Leu | hydrophobic |
| 330666 | *atp6*-2 | exon | 79 | C>U | CCC>UCC | 1 | Pro>Ser | hydrophobic |
| 330796 | a*tp6*-2 | exon | 209 | C>U | CCG>CUG | 2 | Pro>Leu | hydrophobic |
| 330856 | *atp6*-2 | exon | 269 | C>U | UCG>UUG | 2 | Ser>Leu | hydrophobic |
| 330874 | *atp6*-2 | exon | 287 | C>U | UCG>UUG | 2 | Ser>Leu | hydrophobic |
| 330889 | *atp6*-2 | exon | 302 | C>U | CCC>CUU | 2 | Pro>Leu | hydrophobic |
| 330890 | *atp6*-2 | exon | 303 | C>U | CCC>CUU | 2 | Pro>Leu | hydrophobic |
| 331021 | *atp6*-2 | exon | 434 | C>U | UCA>UUA | 2 | Ser>Leu | hydrophobic |
| 331080 | *atp6*-2 | exon | 493 | C>U | CCU>UCU | 1 | Pro>Ser | hydrophobic |
| 331083 | *atp6*-2 | exon | 496 | C>U | CAU>UAU | 1 | His>Tyr | hydrophobic |
| 331105 | *atp6*-2 | exon | 518 | C>U | UCA>UUA | 2 | Ser>Leu | hydrophobic |
| 331147 | *atp6*-2 | exon | 560 | C>U | UCA>UUA | 2 | Ser>Leu | hydrophobic |
| 331168 | *atp6*-2 | exon | 581 | C>U | UCC>UUC | 2 | Ser>Phe | hydrophobic |
| 331255 | *atp6*-2 | exon | 668 | C>U | CCG>CUG | 2 | Pro>Leu | hydrophobic |
| 331276 | *atp6*-2 | exon | 689 | C>U | UCA>UUA | 2 | Ser>Leu | hydrophobic |
| 331284 | *atp6*-2 | exon | 697 | C>U | CAU>UAU | 1 | His>Tyr | hydrophobic |
| 331338 | *atp6*-2 | exon | 751 | C>U | CAA>UAA | 1 | Gln>* | - |
| 338050 | IGR | IGR | - | G>A | - | - | - | - |
| 338194 | IGR | IGR | - | G>U | - | - | - | - |
| 338204 | IGR | IGR | - | U>G | - | - | - | - |
| 338215 | IGR | IGR | - | U>C | - | - | - | - |
| 338220 | IGR | IGR | - | C>U | - | - | - | - |
| 339643 | IGR | IGR | - | G>A | - | - | - | - |
| 340959 | *atp1* | exon | 1029 | G>A | ACC>ACU | 3 | Thr>Thr | - |
| 341742 | *atp1* | exon | 246 | G>A | ACC>ACU | 3 | Thr>Thr | - |
| 342094 | IGR | IGR | - | G>A | - | - | - | - |
| 342136 | IGR | IGR | - | G>A | - | - | - | - |
| 342157 | IGR | IGR | - | G>A | - | - | - | - |
| 342172 | IGR | IGR | - | G>A | - | - | - | - |
| 342181 | IGR | IGR | - | G>A | - | - | - | - |
| 342203 | IGR | IGR | - | G>A | - | - | - | - |
| 342287 | IGR | IGR | - | G>A | - | - | - | - |
| 342297 | IGR | IGR | - | G>A | - | - | - | - |
| 344296 | *ccmFc* | exon | 1327 | G>A | CGA>UGA | 1 | Arg>* | - |
| 344343 | *ccmFc* | exon | 1280 | G>A | UCG>UUG | 2 | Ser>Leu | hydrophobic |
| 344352 | *ccmFc* | exon | 1271 | G>A | UCG>UUG | 2 | Ser>Leu | hydrophobic |
| 344377 | *ccmFc* | exon | 1246 | G>A | CGG>UGG | 1 | Arg>Trp | hydrophobic |
| 344451 | *ccmFc* | exon | 1172 | G>A | UCG>UUG | 2 | Ser>Leu | hydrophobic |
| 344472 | *ccmFc* | exon | 1151 | G>A | CCA>CUA | 2 | Pro>Leu | hydrophobic |
| 344564 | *ccmFc* | exon | 1059 | G>A | CGC>CGU | 3 | Arg>Arg | - |
| 344733 | *ccmFc* | exon | 890 | G>A | UCU>UUU | 2 | Ser>Phe | hydrophobic |
| 344898 | *ccmFc* | Intron | - | G>A | - | - | - | - |
| 348967 | *ccmFc* | exon | 756 | G>A | AUC>AUU | 3 | Ile>Ile | - |
| 349317 | *ccmFc* | exon | 406 | G>A | CGU>UGU | 1 | Arg>Cys | hydrophobic |
| 349389 | *ccmFc* | exon | 334 | G>A | CUU>UUU | 1 | Leu>Phe | Hydrophilic |
| 349402 | *ccmFc* | exon | 321 | G>A | CCC>CCU | 1 | Pro>Pro | - |
| 349413 | *ccmFc* | exon | 310 | G>A | CGU>UGU | 1 | Arg>Cys | hydrophobic |
| 349418 | *ccmFc* | exon | 305 | G>A | UCA>UUA | 2 | Ser>Leu | hydrophobic |
| 349563 | *ccmFc* | exon | 160 | G>A | CCU>UCU | 1 | Pro>Ser | hydrophobic |
| 349572 | *ccmFc* | exon | 151 | G>A | CCU>UCU | 1 | Pro>Ser | hydrophobic |
| 349601 | *ccmFc* | exon | 122 | G>A | UCC>UUC | 2 | Ser>Phe | hydrophobic |
| 349620 | *ccmFc* | exon | 103 | G>A | CCC>UCC | 1 | Pro>Ser | hydrophobic |
| 349671 | *ccmFc* | exon | 52 | G>A | CGU>UGU | 1 | Arg>Cys | hydrophobic |
| 349673 | *ccmFc* | exon | 50 | G>A | CCU>CUU | 2 | Pro>Leu | hydrophobic |
| 349685 | *ccmFc* | exon | 38 | G>A | UCU>UUU | 2 | Ser>Phe | hydrophobic |
| 349816 | IGR | IGR | - | G>A | - | - | - | - |
| 349967 | IGR | IGR | - | G>A | - | - | - | - |
| 350414 | IGR | IGR | - | G>A | - | - | - | - |
| 351690 | *nad1* | exon | 635 | G>A | UCA>UUA | 2 | Ser>Leu | hydrophobic |
| 351745 | *nad1* | exon | 580 | G>A | CGU>UGU | 1 | Arg>Cys | hydrophobic |
| 351752 | *nad1* | exon | 573 | G>A | UUC>UUU | 3 | Phe>Phe | - |
| 351789 | *nad1* | exon | 536 | G>A | UCC>UUU | 2 | Ser>Phe | hydrophobic |
| 351825 | *nad1* | exon | 500 | G>A | UCG>UUG | 2 | Ser>Leu | hydrophobic |
| 351832 | *nad1* | exon | 493 | G>A | CGU>UGU | 1 | Arg>Cys | hydrophobic |
| 351835 | *nad1* | exon | 490 | G>A | CCC>UCU | 1 | Pro>Ser | hydrophobic |
| 353387 | IGR | IGR | - | G>A | - | - | - | - |
| 354306 | IGR | IGR | - | G>A | - | - | - | - |
| 355010 | *cox1* | exon | 1433 | G>A | UCA>UUA | 2 | Ser>Leu | hydrophobic |
| 355038 | *cox1* | exon | 1405 | G>A | CGU>UGU | 1 | Arg>Cys | hydrophobic |
| 355041 | *cox1* | exon | 1402 | G>A | CGU>UGU | 1 | Arg>Cys | hydrophobic |
| 355164 | *cox1* | exon | 1279 | G>A | CUC>UUC | 1 | Leu>Phe | Hydrophilic |
| 355257 | *cox1* | exon | 1186 | G>A | CAC>UAC | 1 | His>Tyr | hydrophobic |
| 355682 | *cox1* | exon | 761 | G>A | UCC>UUC | 2 | Ser>Phe | hydrophobic |
| 355697 | *cox1* | exon | 746 | G>A | CCC>CUC | 2 | Pro>Leu | hydrophobic |
| 355728 | *cox1* | exon | 715 | G>A | CGG>UGG | 1 | Arg>Trp | hydrophobic |
| 355775 | *cox1* | exon | 668 | G>A | UCU>UUU | 2 | Ser>Phe | hydrophobic |
| 355853 | *cox1* | exon | 590 | G>A | CCA>CUA | 2 | Pro>Leu | hydrophobic |
| 355868 | *cox1* | exon | 575 | G>A | CCA>CUA | 2 | Pro>Leu | hydrophobic |
| 355892 | *cox1* | exon | 551 | G>A | UCA>UUA | 2 | Ser>Leu | hydrophobic |
| 355928 | *cox1* | exon | 515 | G>A | UCC>UUC | 2 | Ser>Phe | hydrophobic |
| 355991 | *cox1* | exon | 452 | G>A | UCU>UUU | 2 | Ser>Phe | hydrophobic |
| 356000 | *cox1* | exon | 443 | G>A | UCA>UUA | 2 | Ser>Leu | hydrophobic |
| 356189 | *cox1* | exon | 254 | G>A | UCU>UUU | 2 | Ser>Phe | hydrophobic |
| 356201 | *cox1* | exon | 242 | G>A | UCU>UUU | 2 | Ser>Phe | hydrophobic |
| 356428 | *cox1* | exon | 15 | G>A | GUC>GUU | 3 | Val>Val | - |
| 356432 | *cox1* | exon | 11 | G>A | CCG>CUG | 2 | Pro>Leu | hydrophobic |
| 356674 | *rps10* | exon | 331 | G>A | CGA>UGA | 1 | Arg>* | - |
| 356703 | *rps10* | exon | 302 | G>A | UCG>UUG | 2 | Ser>Leu | hydrophobic |
| 359575 | *rps10* | Intron | - | G>A | - | - | - | - |
| 359596 | *rps10* | exon | 238 | G>A | CGG>UGG | 1 | Arg>Trp | hydrophobic |
| 359600 | *rps10* | exon | 234 | G>A | UUC>UUU | 3 | Phe>Phe | - |
| 359818 | *rps10* | exon | 16 | G>A | CGC>UGC | 1 | Arg>Cys | hydrophobic |
| 359832 | *rps10* | exon | 2 | G>A | ACG>AUG | 2 | Thr>Met | hydrophobic |
| 359882 | IGR | IGR | - | G>A | - | - | - | - |
| 359887 | IGR | IGR | - | G>A | - | - | - | - |
| 360483 | IGR | IGR | - | G>A | - | - | - | - |
| 361184 | *atp4* | exon | 410 | G>A | ACU>AUU | 2 | Thr>Ile | hydrophobic |
| 361193 | *atp4* | exon | 401 | G>A | CCA>CUA | 2 | Pro>Leu | hydrophobic |
| 361205 | *atp4* | exon | 389 | G>A | UCA>UUA | 2 | Ser>Leu | hydrophobic |
| 361294 | *atp4* | exon | 300 | G>A | ACC>ACU | 3 | Thr>Thr | - |
| 361349 | *atp4* | exon | 245 | G>A | CCG>CUG | 2 | Pro>Leu | hydrophobic |
| 361352 | *atp4* | exon | 242 | G>A | CCU>CUU | 2 | Pro>Leu | hydrophobic |
| 361373 | *atp4* | exon | 221 | G>A | CCC>CUC | 2 | Pro>Leu | hydrophobic |
| 361385 | *atp4* | exon | 209 | G>A | UCG>UUG | 2 | Ser>Leu | hydrophobic |
| 361482 | *atp4* | exon | 112 | G>A | CGU>UGU | 1 | Arg>Cys | hydrophobic |
| 361511 | *atp4* | exon | 83 | G>A | UCA>UUA | 2 | Ser>Leu | hydrophobic |
| 361541 | *atp4* | exon | 53 | G>A | UCU>UUU | 2 | Ser>Phe | hydrophobic |
| 361544 | *atp4* | exon | 50 | G>A | CCA>CUA | 2 | Pro>Leu | hydrophobic |
| 361873 | IGR | IGR | - | G>A | - | - | - | - |
| 361882 | IGR | IGR | - | G>A | - | - | - | - |
| 361891 | IGR | IGR | - | G>A | - | - | - | - |
| 361912 | IGR | IGR | - | G>A | - | - | - | - |
| 362660 | IGR | IGR | - | G>A | - | - | - | - |
| 362858 | IGR | IGR | - | G>A | - | - | - | - |
| 363353 | IGR | IGR | - | A>G | - | - | - | - |
| 363460 | *rps12* | exon | 284 | G>A | UCC>UUC | 2 | Ser>Phe | hydrophobic |
| 363523 | *rps12* | exon | 221 | G>A | UCG>UUG | 2 | Ser>Leu | hydrophobic |
| 363548 | *rps12* | exon | 196 | G>A | CAC>UAC | 1 | His>Tyr | hydrophobic |
| 363598 | *rps12* | exon | 146 | G>A | CCA>CUA | 2 | Pro>Leu | hydrophobic |
| 363640 | *rps12* | exon | 104 | G>A | CCG>CUG | 2 | Pro>Leu | hydrophobic |
| 363644 | *rps12* | exon | 100 | G>A | CGC>UGC | 1 | Arg>Cys | hydrophobic |
| 363673 | *rps12* | exon | 71 | G>A | UCG>UUG | 2 | Ser>Leu | hydrophobic |
| 363800 | *nad3* | exon | 349 | G>A | CGG>UGG | 1 | Arg>Trp | hydrophobic |
| 363805 | *nad3* | exon | 344 | G>A | UCG>UUG | 2 | Ser>Leu | hydrophobic |
| 363832 | *nad3* | exon | 317 | G>A | UCU>UUU | 2 | Ser>Phe | hydrophobic |
| 363874 | *nad3* | exon | 275 | G>A | UCU>UUU | 2 | Ser>Phe | hydrophobic |
| 363919 | *nad3* | exon | 230 | G>A | UCC>UUC | 2 | Ser>Phe | hydrophobic |
| 363934 | *nad3* | exon | 215 | G>A | CCG>CUG | 2 | Pro>Leu | hydrophobic |
| 363940 | *nad3* | exon | 209 | G>A | CCU>UUU | 2 | Pro>Phe | hydrophobic |
| 363941 | *nad3* | exon | 208 | G>A | CCU>UUU | 1 | Pro>Phe | hydrophobic |
| 364069 | *nad3* | exon | 80 | G>A | CCC>CUC | 2 | Pro>Leu | hydrophobic |
| 364144 | *nad3* | exon | 5 | G>A | UCA>UUA | 2 | Ser>Leu | hydrophobic |
| 364581 | IGR | IGR | - | G>A | - | - | - | - |
| 364806 | *trnY-GUA* | tRNA | 72 | G>A |  | 2 |  |  |
| 385093 | *cob* | exon | 1118 | G>A | CCG>CUG | 2 | Pro>Leu | hydrophobic |
| 385133 | *cob* | exon | 1078 | G>A | CCU>UCU | 1 | Pro>Ser | hydrophobic |
| 385136 | *cob* | exon | 1075 | G>A | CCU>UCU | 1 | Pro>Ser | hydrophobic |
| 385202 | *cob* | exon | 1009 | G>A | CGC>UGC | 1 | Arg>Cys | hydrophobic |
| 385235 | *cob* | exon | 976 | G>A | CAC>UAC | 1 | His>Tyr | hydrophobic |
| 385309 | *cob* | exon | 902 | G>A | CCA>CUA | 2 | Pro>Leu | hydrophobic |
| 385364 | *cob* | exon | 847 | G>A | CAU>UAU | 1 | His>Tyr | hydrophobic |
| 385409 | *cob* | exon | 802 | G>A | CCC>UCC | 1 | Pro>Ser | hydrophobic |
| 385426 | *cob* | exon | 388 | G>A | ACA>AUA | 2 | Thr>Ile | hydrophobic |
| 385649 | *cob* | exon | 562 | G>A | CAU>UAU | 1 | His>Tyr | hydrophobic |
| 385810 | *cob* | exon | 401 | G>A | ACA>AUA | 2 | Thr>Ile | hydrophobic |
| 385859 | *cob* | exon | 352 | G>A | CGG>UGG | 1 | Arg>Trp | hydrophobic |
| 385892 | *cob* | exon | 319 | G>A | CAU>UAU | 1 | His>Tyr | hydrophobic |
| 385919 | *cob* | exon | 292 | G>A | CAC>UAC | 1 | His>Tyr | hydrophobic |
| 385931 | *cob* | exon | 280 | G>A | CUC>UUC | 1 | Leu>Phe | Hydrophilic |
| 386098 | *cob* | exon | 113 | G>A | UCG>UUG | 2 | Ser>Leu | hydrophobic |
| 386170 | *cob* | exon | 41 | G>A | UCC>UUC | 2 | Ser>Phe | hydrophobic |
| 387162 | *rps14* | exon | 271 | G>A | CCU>UCU | 1 | Pro>Ser | hydrophobic |
| 387239 | *rps14* | exon | 194 | G>A | UCC>UUC | 2 | Ser>Phe | hydrophobic |
| 387423 | *rps14* | exon | 10 | G>A | CAG>UAG | 1 | Gln>* | - |
| 387483 | *rpl5* | exon | 515 | G>A | CCG>CUG | 2 | Pro>Leu | hydrophobic |
| 387486 | *rpl5* | exon | 512 | G>A | CCA>CUA | 2 | Pro>Leu | hydrophobic |
| 387669 | *rpl5* | exon | 329 | G>A | UCG>UUG | 2 | Ser>Leu | hydrophobic |
| 387681 | *rpl5* | exon | 317 | G>A | UCG>UUG | 2 | Ser>Leu | hydrophobic |
| 387829 | *rpl5* | exon | 169 | G>A | CGC>UGC | 1 | Arg>Cys | hydrophobic |
| 387906 | *rpl5* | exon | 92 | G>A | UCG>UUG | 2 | Ser>Leu | hydrophobic |
| 387934 | *rpl5* | exon | 64 | G>A | CAC>UAC | 1 | His>Tyr | hydrophobic |
| 387951 | *rpl5* | exon | 47 | G>A | CCG>CUG | 2 | Pro>Leu | hydrophobic |
| 387963 | *rpl5* | exon | 35 | G>A | UCA>UUA | 2 | Ser>Leu | hydrophobic |
| 388006 | IGR | IGR | - | G>A | - | - | - | - |
| 388787 | IGR | IGR | - | G>A | - | - | - | - |
| 388891 | IGR | IGR | - | G>A | - | - | - | - |
| 389237 | IGR | IGR | - | G>A | - | - | - | - |
| 394711 | IGR | IGR | - | G>A | - | - | - | - |
| 395092 | IGR | IGR | - | G>A | - | - | - | - |
| 395401 | IGR | IGR | - | G>A | - | - | - | - |
| 395494 | *cox3* | exon | 754 | G>A | CGG>UGG | 1 | Arg>Trp | hydrophobic |
| 395645 | *cox3* | exon | 603 | G>A | UUC>UUU | 3 | Phe>Ser | - |
| 395736 | *cox3* | exon | 512 | G>A | UCA>UUA | 2 | Ser>Leu | hydrophobic |
| 395835 | *cox3* | exon | 413 | G>A | CCU>CUU | 2 | Pro>Leu | hydrophobic |
| 395860 | *cox3* | exon | 388 | G>A | CGG>UGG | 1 | Arg>Trp | hydrophobic |
| 395934 | *cox3* | exon | 314 | G>A | UCU>UUU | 2 | Ser>Phe | hydrophobic |
| 395937 | *cox3* | exon | 311 | G>A | UCU>UUU | 2 | Ser>Phe | hydrophobic |
| 395944 | *cox3* | exon | 304 | G>A | CGG>UGG | 1 | Arg>Trp | hydrophobic |
| 395959 | *cox3* | exon | 289 | G>A | CUU>UUU | 1 | Leu>Phe | Hydrophilic |
| 395985 | *cox3* | exon | 263 | G>A | CCG>CUG | 2 | Pro>Leu | hydrophobic |
| 395991 | *cox3* | exon | 257 | G>A | UCU>UUU | 2 | Ser>Phe | hydrophobic |
| 396003 | *cox3* | exon | 245 | G>A | CCU>CUU | 2 | Pro>Leu | hydrophobic |
| 396606 | IGR | IGR | - | G>A | - | - | - | - |
| 414588 | IGR | IGR | - | C>U | - | - | - | - |
| 414882 | *nad4L* | exon | 2 | C>U | ACG>AUG | 2 | Thr>Met | hydrophobic |
| 414921 | *nad4L* | exon | 41 | C>U | UCU>UUU | 2 | Ser>Phe | hydrophobic |
| 414935 | *nad4L* | exon | 55 | C>U | CGG>UGG | 1 | Arg>Trp | hydrophobic |
| 414966 | *nad4L* | exon | 86 | C>U | CCU>CUU | 2 | Pro>Leu | hydrophobic |
| 415011 | *nad4L* | exon | 131 | C>U | UCG>UUG | 2 | Ser>Leu | hydrophobic |
| 415038 | *nad4L* | exon | 158 | C>U | UCG>UUG | 2 | Ser>Leu | hydrophobic |
| 415059 | *nad4L* | exon | 179 | C>U | UCA>UUA | 2 | Ser>Leu | hydrophobic |
| 415068 | *nad4L* | exon | 188 | C>U | UCA>UUA | 2 | Ser>Leu | hydrophobic |
| 415077 | *nad4L* | exon | 197 | C>U | UCA>UUA | 2 | Ser>Leu | hydrophobic |
| 415406 | IGR | IGR | - | C>U | - | - | - | - |
| 428477 | *nad6* | exon | 463 | G>A | CCU>UCU | 1 | Pro>Ser | hydrophobic |
| 428651 | *nad6* | exon | 289 | G>A | CUU>UUU | 1 | Leu>Phe | Hydrophilic |
| 428749 | *nad6* | exon | 191 | G>A | UCA>UUA | 2 | Ser>Leu | hydrophobic |
| 428771 | *nad6* | exon | 169 | G>A | CAU>UAU | 1 | His>Tyr | hydrophobic |
| 428779 | *nad6* | exon | 161 | G>A | CCA>CUA | 2 | Pro>Leu | hydrophobic |
| 428794 | *nad6* | exon | 146 | G>A | UCC>UUC | 2 | Ser>Phe | hydrophobic |
| 428837 | *nad6* | exon | 103 | G>A | CGC>UGC | 1 | Arg>Cys | hydrophobic |
| 428851 | *nad6* | exon | 89 | G>A | CCC>UUC | 2 | Pro>Phe | hydrophobic |
| 428852 | *nad6* | exon | 88 | G>A | CCC>UUC | 1 | Pro>Phe | hydrophobic |
| 428887 | *nad6* | exon | 53 | G>A | GCA>GUA | 2 | Ala>Val | hydrophobic |
| 428914 | *nad6* | exon | 26 | G>A | CCU>CUU | 2 | Pro>Leu | hydrophobic |
| 432290 | *nad1* | exon | 368 | G>A | ACA>AUA | 2 | Thr>Ile | hydrophobic |
| 432350 | *nad1* | exon | 308 | G>A | UCG>UUG | 2 | Ser>Leu | hydrophobic |
| 432393 | *nad1* | exon | 265 | G>A | CGG>UGG | 1 | Arg>Trp | hydrophobic |
| 432443 | *nad1* | exon | 215 | G>A | UCC>UUC | 2 | Ser>Phe | hydrophobic |
| 432656 | *nad1* | exon | 2 | G>A | ACG>AUG | 2 | Thr>Met | hydrophobic |
| 432756 | IGR | IGR | - | G>A | - | - | - | - |
| 432791 | IGR | IGR | - | G>A | - | - | - | - |
| 432980 | IGR | IGR | - | G>A | - | - | - | - |
| 433072 | IGR | IGR | - | G>A | - | - | - | - |
| 433228 | IGR | IGR | - | G>A | - | - | - | - |
| 433306 | IGR | IGR | - | G>A | - | - | - | - |
| 433388 | *ccmB* | exon | 596 | G>A | GCG>GUG | 2 | Ala>Val | hydrophobic |
| 433412 | *ccmB* | exon | 572 | G>A | CCG>CUG | 2 | Pro>Leu | hydrophobic |
| 433415 | *ccmB* | exon | 569 | G>A | UCU>UUU | 2 | Ser>Phe | hydrophobic |
| 433418 | *ccmB* | exon | 566 | G>A | UCC>UUC | 2 | Ser>Phe | hydrophobic |
| 433430 | *ccmB* | exon | 554 | G>A | UCG>UUG | 2 | Ser>Leu | hydrophobic |
| 433433 | *ccmB* | exon | 551 | G>A | UCA>UUA | 2 | Ser>Leu | hydrophobic |
| 433508 | *ccmB* | exon | 476 | G>A | CCA>UUA | 2 | Pro>Leu | hydrophobic |
| 433509 | *ccmB* | exon | 475 | G>A | CCA>UUA | 1 | Pro>Leu | hydrophobic |
| 433517 | *ccmB* | exon | 467 | G>A | UCG>UUG | 2 | Ser>Leu | hydrophobic |
| 433560 | *ccmB* | exon | 424 | G>A | CGU>UGU | 1 | Arg>Cys | hydrophobic |
| 433604 | *ccmB* | exon | 380 | G>A | CCA>CUA | 2 | Pro>Leu | hydrophobic |
| 433617 | *ccmB* | exon | 367 | G>A | CGG>UGG | 1 | Arg>Trp | hydrophobic |
| 433646 | *ccmB* | exon | 338 | G>A | CCG>CUG | 2 | Pro>Leu | hydrophobic |
| 433698 | *ccmB* | exon | 286 | G>A | CGG>UGG | 1 | Arg>Trp | hydrophobic |
| 433790 | *ccmB* | exon | 194 | G>A | CCU>CUU | 2 | Pro>Leu | hydrophobic |
| 433824 | *ccmB* | exon | 160 | G>A | CCU>UCU | 1 | Pro>Ser | hydrophobic |
| 433836 | *ccmB* | exon | 148 | G>A | CCG>UCG | 1 | Pro>Ser | hydrophobic |
| 433856 | *ccmB* | exon | 128 | G>A | UCA>UUA | 2 | Ser>Leu | hydrophobic |
| 433897 | *ccmB* | exon | 87 | G>A | AUC>AUU | 3 | Ile>Phe | - |
| 433913 | *ccmB* | exon | 71 | G>A | CCA>CUA | 2 | Pro>Leu | hydrophobic |
| 433941 | *ccmB* | exon | 43 | G>A | CCC>UCC | 1 | Pro>Ser | hydrophobic |
| 438557 | IGR | IGR | - | C>U | - | - | - | - |
| 438621 | IGR | IGR | - | C>U | - | - | - | - |
| 439020 | IGR | IGR | - | C>U | - | - | - | - |
| 439164 | *nad7* | exon | 38 | C>U | UCG>UUG | 2 | Ser>Leu | hydrophobic |
| 439171 | *nad7* | exon | 45 | C>U | UUC>UUU | 3 | Phe>Phe | - |
| 439203 | *nad7* | exon | 77 | C>U | UCA>UUA | 2 | Ser>Leu | hydrophobic |
| 439209 | *nad7* | exon | 83 | C>U | UCA>UUA | 2 | Ser>Leu | hydrophobic |
| 439263 | *nad7* | exon | 137 | C>U | UCA>UUA | 2 | Ser>Leu | hydrophobic |
| 440231 | *nad7* | exon | 200 | C>U | UCU>UUU | 2 | Ser>Phe | hydrophobic |
| 441567 | *nad7* | Intron | - | C>U | - | - | - | - |
| 441587 | *nad7* | exon | 224 | C>U | ACG>AUG | 2 | Thr>Met | hydrophobic |
| 441607 | *nad7* | exon | 244 | C>U | CAU>UAU | 1 | His>Tyr | hydrophobic |
| 441614 | *nad7* | exon | 251 | C>U | UCA>UUA | 2 | Ser>Leu | hydrophobic |
| 441679 | *nad7* | exon | 316 | C>U | CGU>UGU | 1 | Arg>Cys | hydrophobic |
| 441698 | *nad7* | exon | 335 | C>U | UCA>UUA | 2 | Ser>Leu | hydrophobic |
| 441746 | *nad7* | exon | 383 | C>U | UCA>UUA | 2 | Ser>Leu | hydrophobic |
| 441755 | *nad7* | exon | 392 | C>U | UCC>UUC | 2 | Ser>Phe | hydrophobic |
| 441941 | *nad7* | exon | 578 | C>U | UCA>UUA | 2 | Ser>Phe | hydrophobic |
| 443116 | *nad7* | exon | 698 | C>U | UCG>UUG | 2 | Ser>Leu | hydrophobic |
| 443142 | *nad7* | exon | 724 | C>U | CAU>UAU | 1 | His>Tyr | hydrophobic |
| 443157 | *nad7* | exon | 739 | C>U | CCU>UUU | 1 | Pro>Phe | hydrophobic |
| 443158 | *nad7* | exon | 740 | C>U | CCU>UUU | 2 | Pro>Phe | hydrophobic |
| 443187 | *nad7* | exon | 769 | C>U | CGC>UGC | 1 | Arg>Cys | hydrophobic |
| 443254 | *nad7* | exon | 836 | C>U | CCU>CUU | 2 | Pro>Leu | hydrophobic |
| 445243 | *nad7* | Intron | - | C>U | - | - | - | - |
| 445414 | *nad7* | exon | 963 | C>U | UCC>UCU | 3 | Ser>Leu | - |
| 445424 | *nad7* | exon | 973 | C>U | CCU>UCU | 1 | Pro>Ser | hydrophobic |
| 445508 | *nad7* | exon | 1057 | C>U | CGU>UGU | 1 | Arg>Cys | hydrophobic |
| 445530 | *nad7* | exon | 1079 | C>U | UCU>UUU | 2 | Ser>Phe | hydrophobic |
| 445539 | *nad7* | exon | 1088 | C>U | UCA>UUA | 2 | Ser>Leu | hydrophobic |
| 445554 | *nad7* | exon | 1103 | C>U | UCU>UUU | 2 | Ser>Phe | hydrophobic |
| 445575 | *nad7* | exon | 1124 | C>U | CCA>CUA | 2 | Pro>Leu | hydrophobic |
| 445617 | *nad7* | exon | 1166 | C>U | UCU>UUU | 2 | Ser>Phe | hydrophobic |
| 445969 | *IGR* | IGR | - | C>U | - | - | - | - |
| 447744 | *nad5* | exon | 1490 | C>U | CCC>CUC | 2 | Pro>Leu | hydrophobic |
| 447804 | *nad5* | exon | 1550 | C>U | ACC>AUC | 2 | Thr>Ile | hydrophobic |
| 447834 | *nad5* | exon | 1580 | C>U | UCA>UUA | 2 | Ser>Leu | hydrophobic |
| 447843 | *nad5* | exon | 1589 | C>U | UCU>UUU | 2 | Ser>Phe | hydrophobic |
| 447864 | *nad5* | exon | 1610 | C>U | CCC>CUC | 2 | Pro>Leu | hydrophobic |
| 447919 | *nad5* | exon | 1665 | C>U | CUC>CUU | 3 | Leu>Leu | - |
| 449069 | *nad5* | exon | 1895 | C>U | UCA>UUA | 2 | Ser>Leu | hydrophobic |
| 439493 | IGR | - | - | C>U | - | - | - | - |
| 449092 | *nad5* | exon | 1918 | C>U | CGU>UGU | 1 | Arg>Cys | hydrophobic |

**Supplementary Table 4 Basic clustering of soybean PPR gene family.**

| Subfamily | No.of member | Member |
| --- | --- | --- |
| P | 469 | Gm_DFD3_00044.t01;Gm_DFD3_00154.t01;Gm_DFD3_00208.t01;Gm_DFD3_00521.t01;Gm_DFD3_00526.t01;Gm_DFD3_00530.t01;Gm_DFD3_00532.t01;Gm_DFD3_00537.t01;Gm_DFD3_00639.t01;Gm_DFD3_00826.t01;Gm_DFD3_00883.t01;Gm_DFD3_00884.t01;Gm_DFD3_01135.t01;Gm_DFD3_01191.t01;Gm_DFD3_01257.t01;Gm_DFD3_01285.t01;Gm_DFD3_01468.t01;Gm_DFD3_01489.t01;Gm_DFD3_01490.t01;Gm_DFD3_01700.t01;Gm_DFD3_01960.t01;Gm_DFD3_01978.t01;Gm_DFD3_01986.t01;Gm_DFD3_02009.t01;Gm_DFD3_02128.t01;Gm_DFD3_02150.t01;Gm_DFD3_02215.t01;Gm_DFD3_02228.t01;Gm_DFD3_02912.t01;Gm_DFD3_03174.t01;Gm_DFD3_03177.t01;Gm_DFD3_03182.t01;Gm_DFD3_03184.t01;Gm_DFD3_03367.t01;Gm_DFD3_03805.t01;Gm_DFD3_03907.t01;Gm_DFD3_04007.t01;Gm_DFD3_04245.t01;Gm_DFD3_04501.t01;Gm_DFD3_04545.t03;Gm_DFD3_04585.t01;Gm_DFD3_04601.t01;Gm_DFD3_04626.t01;Gm_DFD3_04633.t01;Gm_DFD3_04862.t01;Gm_DFD3_05083.t01;Gm_DFD3_05138.t01;Gm_DFD3_05233.t01;Gm_DFD3_05527.t01;Gm_DFD3_05753.t02;Gm_DFD3_05755.t01;Gm_DFD3_05904.t01;Gm_DFD3_05986.t01;Gm_DFD3_06090.t01;Gm_DFD3_06160.t01;Gm_DFD3_06207.t01;Gm_DFD3_06460.t01;Gm_DFD3_06473.t01;Gm_DFD3_06526.t01;Gm_DFD3_06795.t01;Gm_DFD3_07056.t01;Gm_DFD3_07369.t01;Gm_DFD3_07379.t01;Gm_DFD3_07562.t01;Gm_DFD3_07714.t01;Gm_DFD3_07766.t01;Gm_DFD3_08041.t01;Gm_DFD3_08055.t01;Gm_DFD3_08507.t01;Gm_DFD3_08592.t01;Gm_DFD3_08650.t02;Gm_DFD3_08890.t01;Gm_DFD3_08956.t01;Gm_DFD3_08957.t01;Gm_DFD3_09043.t01;Gm_DFD3_09186.t01;Gm_DFD3_09251.t01;Gm_DFD3_09253.t01;Gm_DFD3_09333.t01;Gm_DFD3_09421.t01;Gm_DFD3_09613.t01;Gm_DFD3_09672.t01;Gm_DFD3_09686.t01;Gm_DFD3_09952.t01;Gm_DFD3_10050.t01;Gm_DFD3_10289.t01;Gm_DFD3_10290.t01;Gm_DFD3_10349.t01;Gm_DFD3_10383.t01;Gm_DFD3_10510.t01;Gm_DFD3_10609.t01;Gm_DFD3_10700.t01;Gm_DFD3_10908.t01;Gm_DFD3_10928.t01;Gm_DFD3_10990.t01;Gm_DFD3_10992.t01;Gm_DFD3_11403.t01;Gm_DFD3_11426.t01;Gm_DFD3_11572.t01;Gm_DFD3_11771.t01;Gm_DFD3_11781.t02;Gm_DFD3_11795.t01;Gm_DFD3_11916.t01;Gm_DFD3_12156.t01;Gm_DFD3_12337.t01;Gm_DFD3_12449.t01;Gm_DFD3_12453.t01;Gm_DFD3_12678.t01;Gm_DFD3_12704.t01;Gm_DFD3_12705.t01;Gm_DFD3_12790.t01;Gm_DFD3_12939.t01;Gm_DFD3_13352.t01;Gm_DFD3_13414.t01;Gm_DFD3_13636.t01;Gm_DFD3_13765.t01;Gm_DFD3_13925.t01;Gm_DFD3_14082.t01;Gm_DFD3_14236.t01;Gm_DFD3_14727.t01;Gm_DFD3_14958.t01;Gm_DFD3_15088.t01;Gm_DFD3_15343.t01;Gm_DFD3_15368.t01;Gm_DFD3_15376.t01;Gm_DFD3_15383.t01;Gm_DFD3_15418.t01;Gm_DFD3_15432.t01;Gm_DFD3_15450.t01;Gm_DFD3_15529.t01;Gm_DFD3_15633.t01;Gm_DFD3_15749.t01;Gm_DFD3_15762.t01;Gm_DFD3_15877.t01;Gm_DFD3_15886.t01;Gm_DFD3_15910.t01;Gm_DFD3_15963.t01;Gm_DFD3_15996.t01;Gm_DFD3_16010.t02;Gm_DFD3_16085.t01;Gm_DFD3_16092.t01;Gm_DFD3_16151.t01;Gm_DFD3_16193.t01;Gm_DFD3_16194.t01;Gm_DFD3_16366.t01;Gm_DFD3_16374.t01;Gm_DFD3_16379.t01;Gm_DFD3_16382.t01;Gm_DFD3_17220.t01;Gm_DFD3_17247.t01;Gm_DFD3_17378.t01;Gm_DFD3_17385.t01;Gm_DFD3_17391.t01;Gm_DFD3_17453.t01;Gm_DFD3_17461.t01;Gm_DFD3_17734.t01;Gm_DFD3_17804.t01;Gm_DFD3_17900.t01;Gm_DFD3_18121.t01;Gm_DFD3_18206.t01;Gm_DFD3_18511.t01;Gm_DFD3_18553.t01;Gm_DFD3_18647.t01;Gm_DFD3_18763.t01;Gm_DFD3_18855.t01;Gm_DFD3_19080.t01;Gm_DFD3_19119.t01;Gm_DFD3_19120.t01;Gm_DFD3_19122.t01;Gm_DFD3_19254.t01;Gm_DFD3_19255.t01;Gm_DFD3_19268.t01;Gm_DFD3_19382.t01;Gm_DFD3_19485.t01;Gm_DFD3_19506.t01;Gm_DFD3_19545.t01;Gm_DFD3_19560.t01;Gm_DFD3_19588.t01;Gm_DFD3_20098.t01;Gm_DFD3_21690.t01;Gm_DFD3_21692.t01;Gm_DFD3_21694.t01;Gm_DFD3_21696.t01;Gm_DFD3_21705.t01;Gm_DFD3_21707.t01;Gm_DFD3_21723.t01;Gm_DFD3_22570.t01;Gm_DFD3_22700.t01;Gm_DFD3_22749.t01;Gm_DFD3_22771.t01;Gm_DFD3_22820.t01;Gm_DFD3_22843.t01;Gm_DFD3_22858.t01;Gm_DFD3_22992.t01;Gm_DFD3_23155.t01;Gm_DFD3_23205.t01;Gm_DFD3_23493.t01;Gm_DFD3_23567.t01;Gm_DFD3_23870.t01;Gm_DFD3_23978.t01;Gm_DFD3_24131.t01;Gm_DFD3_24167.t01;Gm_DFD3_24168.t01;Gm_DFD3_24421.t01;Gm_DFD3_24613.t01;Gm_DFD3_24818.t01;Gm_DFD3_25090.t01;Gm_DFD3_25098.t01;Gm_DFD3_25206.t01;Gm_DFD3_25269.t01;Gm_DFD3_25270.t01;Gm_DFD3_25273.t01;Gm_DFD3_25408.t01;Gm_DFD3_25478.t01;Gm_DFD3_25502.t01;Gm_DFD3_25517.t01;Gm_DFD3_25519.t01;Gm_DFD3_25532.t01;Gm_DFD3_25964.t01;Gm_DFD3_25999.t01;Gm_DFD3_26001.t04;Gm_DFD3_26122.t01;Gm_DFD3_26129.t01;Gm_DFD3_26188.t01;Gm_DFD3_26199.t01;Gm_DFD3_26318.t01;Gm_DFD3_26356.t01;Gm_DFD3_26377.t01;Gm_DFD3_26426.t01;Gm_DFD3_26432.t01;Gm_DFD3_26530.t01;Gm_DFD3_26644.t01;Gm_DFD3_26646.t01;Gm_DFD3_26930.t01;Gm_DFD3_27150.t01;Gm_DFD3_27438.t01;Gm_DFD3_27784.t01;Gm_DFD3_27808.t01;Gm_DFD3_28050.t01;Gm_DFD3_28102.t01;Gm_DFD3_28161.t01;Gm_DFD3_28249.t01;Gm_DFD3_28263.t01;Gm_DFD3_28360.t01;Gm_DFD3_28372.t01;Gm_DFD3_28437.t01;Gm_DFD3_28475.t01;Gm_DFD3_28609.t01;Gm_DFD3_28874.t01;Gm_DFD3_28875.t01;Gm_DFD3_28998.t02;Gm_DFD3_29231.t02;Gm_DFD3_29440.t01;Gm_DFD3_29441.t01;Gm_DFD3_29476.t01;Gm_DFD3_29567.t01;Gm_DFD3_29590.t01;Gm_DFD3_29629.t01;Gm_DFD3_30131.t01;Gm_DFD3_30373.t01;Gm_DFD3_30902.t01;Gm_DFD3_31038.t01;Gm_DFD3_31419.t01;Gm_DFD3_31525.t01;Gm_DFD3_31526.t01;Gm_DFD3_31666.t01;Gm_DFD3_31870.t01;Gm_DFD3_31895.t01;Gm_DFD3_31903.t01;Gm_DFD3_31953.t01;Gm_DFD3_32029.t01;Gm_DFD3_32381.t02;Gm_DFD3_32615.t01;Gm_DFD3_32646.t01;Gm_DFD3_32764.t01;Gm_DFD3_32802.t01;Gm_DFD3_32803.t01;Gm_DFD3_32987.t01;Gm_DFD3_33038.t01;Gm_DFD3_33161.t01;Gm_DFD3_33186.t01;Gm_DFD3_33189.t01;Gm_DFD3_33214.t01;Gm_DFD3_33262.t01;Gm_DFD3_33296.t01;Gm_DFD3_33325.t01;Gm_DFD3_33422.t02;Gm_DFD3_33492.t01;Gm_DFD3_33661.t01;Gm_DFD3_33679.t01;Gm_DFD3_33722.t01;Gm_DFD3_33773.t01;Gm_DFD3_34044.t01;Gm_DFD3_34527.t01;Gm_DFD3_34565.t01;Gm_DFD3_34568.t01;Gm_DFD3_34675.t01;Gm_DFD3_34929.t01;Gm_DFD3_34980.t01;Gm_DFD3_35094.t01;Gm_DFD3_35190.t01;Gm_DFD3_35284.t01;Gm_DFD3_35295.t01;Gm_DFD3_35330.t01;Gm_DFD3_35395.t01;Gm_DFD3_35474.t01;Gm_DFD3_35499.t01;Gm_DFD3_35676.t01;Gm_DFD3_35843.t01;Gm_DFD3_36005.t01;Gm_DFD3_36074.t01;Gm_DFD3_36162.t01;Gm_DFD3_36171.t01;Gm_DFD3_36174.t01;Gm_DFD3_36194.t01;Gm_DFD3_36195.t01;Gm_DFD3_36314.t01;Gm_DFD3_36377.t01;Gm_DFD3_36418.t01;Gm_DFD3_36464.t01;Gm_DFD3_36809.t01;Gm_DFD3_37114.t01;Gm_DFD3_37127.t01;Gm_DFD3_37370.t01;Gm_DFD3_37445.t01;Gm_DFD3_37509.t01;Gm_DFD3_37588.t01;Gm_DFD3_37634.t06;Gm_DFD3_37706.t01;Gm_DFD3_37991.t01;Gm_DFD3_38094.t01;Gm_DFD3_38107.t01;Gm_DFD3_38178.t01;Gm_DFD3_38190.t01;Gm_DFD3_38218.t01;Gm_DFD3_38232.t01;Gm_DFD3_38234.t01;Gm_DFD3_38593.t01;Gm_DFD3_38647.t01;Gm_DFD3_38880.t01;Gm_DFD3_38997.t01;Gm_DFD3_39062.t01;Gm_DFD3_39064.t01;Gm_DFD3_39077.t01;Gm_DFD3_39078.t01;Gm_DFD3_39080.t01;Gm_DFD3_39086.t01;Gm_DFD3_39087.t02;Gm_DFD3_39113.t03;Gm_DFD3_39347.t01;Gm_DFD3_39348.t01;Gm_DFD3_39349.t01;Gm_DFD3_39354.t02;Gm_DFD3_39368.t01;Gm_DFD3_39384.t01;Gm_DFD3_39445.t01;Gm_DFD3_39555.t01;Gm_DFD3_39742.t01;Gm_DFD3_39909.t01;Gm_DFD3_39954.t01;Gm_DFD3_40048.t01;Gm_DFD3_40476.t01;Gm_DFD3_40525.t01;Gm_DFD3_40551.t01;Gm_DFD3_40957.t01;Gm_DFD3_41029.t01;Gm_DFD3_41090.t01;Gm_DFD3_41292.t01;Gm_DFD3_41387.t01;Gm_DFD3_41406.t01;Gm_DFD3_41518.t01;Gm_DFD3_41818.t01;Gm_DFD3_41932.t01;Gm_DFD3_41959.t01;Gm_DFD3_42298.t01;Gm_DFD3_42336.t01;Gm_DFD3_42375.t01;Gm_DFD3_42738.t01;Gm_DFD3_42856.t01;Gm_DFD3_42857.t01;Gm_DFD3_43006.t01;Gm_DFD3_43377.t01;Gm_DFD3_43503.t01;Gm_DFD3_43515.t01;Gm_DFD3_43600.t01;Gm_DFD3_43619.t01;Gm_DFD3_43722.t01;Gm_DFD3_43790.t01;Gm_DFD3_43800.t01;Gm_DFD3_43982.t01;Gm_DFD3_44085.t01;Gm_DFD3_44103.t01;Gm_DFD3_44226.t01;Gm_DFD3_44227.t01;Gm_DFD3_44241.t01;Gm_DFD3_44407.t01;Gm_DFD3_44477.t01;Gm_DFD3_44569.t01;Gm_DFD3_44649.t01;Gm_DFD3_44863.t01;Gm_DFD3_44919.t01;Gm_DFD3_45194.t01;Gm_DFD3_45274.t01;Gm_DFD3_45334.t01;Gm_DFD3_45346.t01;Gm_DFD3_45421.t01;Gm_DFD3_45606.t01;Gm_DFD3_45727.t01;Gm_DFD3_45731.t01;Gm_DFD3_46090.t01;Gm_DFD3_46104.t01;Gm_DFD3_46105.t01;Gm_DFD3_46654.t01;Gm_DFD3_46859.t01;Gm_DFD3_46880.t01;Gm_DFD3_46883.t01;Gm_DFD3_46886.t01;Gm_DFD3_46923.t01;Gm_DFD3_46998.t02;Gm_DFD3_47140.t01;Gm_DFD3_47311.t01;Gm_DFD3_47401.t01;Gm_DFD3_47454.t02;Gm_DFD3_47477.t01Gm_DFD3_47504.t01;Gm_DFD3_47521.t01;Gm_DFD3_47584.t01;Gm_DFD3_47587.t01;Gm_DFD3_47600.t01;Gm_DFD3_47678.t01;Gm_DFD3_47777.t01;Gm_DFD3_47830.t01;Gm_DFD3_48048.t01;Gm_DFD3_48088.t01;Gm_DFD3_48436.t01;Gm_DFD3_48467.t01;Gm_DFD3_48663.t03;Gm_DFD3_48681.t01;Gm_DFD3_48682.t01’Gm_DFD3_48704.t01;Gm_DFD3_35444.t01;Gm_DFD3_21693.t01;Gm_DFD3_22459.t01;Gm_DFD3_21662.t01;Gm_DFD3_20757.t01;Gm_DFD3_25267.t01;Gm_DFD3_21689.t01;Gm_DFD3_23246.t01;Gm_DFD3_21544.t01;Gm_DFD3_20713.t01;Gm_DFD3_21686.t01;Gm_DFD3_22339.t01;Gm_DFD3_22528.t01;Gm_DFD3_22632.t01;Gm_DFD3_22262.t01;Gm_DFD3_21528.t01;Gm_DFD3_22674.t01;Gm_DFD3_22321.t02;Gm_DFD3_21644.t01;Gm_DFD3_21704.t01;Gm_DFD3_21643.t01;Gm_DFD3_21885.t01;Gm_DFD3_21699.t01;Gm_DFD3_21931.t01;Gm_DFD3_21708.t01;Gm_DFD3_20973.t01;Gm_DFD3_20489.t01;Gm_DFD3_21024.t01;Gm_DFD3_21725.t01 |
| PLS | 39 | Gm_DFD3_04755.t01;Gm_DFD3_10369.t01;Gm_DFD3_16830.t01;Gm_DFD3_18534.t01;Gm_DFD3_17884.t01;Gm_DFD3_23795.t01;Gm_DFD3_22757.t01;Gm_DFD3_25463.t01;Gm_DFD3_25978.t01;Gm_DFD3_29684.t01;Gm_DFD3_25518.t01;Gm_DFD3_28129.t01;Gm_DFD3_31669.t01;Gm_DFD3_32513.t01;Gm_DFD3_36548.t01;Gm_DFD3_36420.t01;Gm_DFD3_36830.t01;Gm_DFD3_34381.t01;Gm_DFD3_33491.t01;Gm_DFD3_37373.t01;Gm_DFD3_36419.t01;Gm_DFD3_40757.t01;Gm_DFD3_39650.t01;Gm_DFD3_38301.t01;Gm_DFD3_40380.t01;Gm_DFD3_45136.t01;Gm_DFD3_45528.t01;Gm_DFD3_44943.t01;Gm_DFD3_47350.t01;Gm_DFD3_45979.t01;Gm_DFD3_47633.t06;Gm_DFD3_02028.t01;Gm_DFD3_00533.t03;Gm_DFD3_20372.t01;Gm_DFD3_20371.t01;Gm_DFD3_21670.t01;Gm_DFD3_20370.t01;Gm_DFD3_22106.t01;Gm_DFD3_20369.t01; |
| E+ | 125 | Gm_DFD3_00500.t01;Gm_DFD3_00511.t01;Gm_DFD3_01126.t01;Gm_DFD3_01245.t01;Gm_DFD3_01298.t01;Gm_DFD3_01343.t01;Gm_DFD3_01434.t01;Gm_DFD3_01466.t01;Gm_DFD3_01505.t01;Gm_DFD3_01953.t01;Gm_DFD3_02118.t01;Gm_DFD3_02187.t01;Gm_DFD3_02313.t01;Gm_DFD3_02536.t01;Gm_DFD3_02830.t01;Gm_DFD3_02916.t01;Gm_DFD3_03162.t01;Gm_DFD3_03733.t01;Gm_DFD3_04026.t01;Gm_DFD3_04068.t01;Gm_DFD3_04312.t01;Gm_DFD3_04888.t01;Gm_DFD3_04896.t01;Gm_DFD3_05125.t01;Gm_DFD3_05574.t01;Gm_DFD3_06085.t01;Gm_DFD3_06366.t02;Gm_DFD3_06419.t01;Gm_DFD3_06933.t01;Gm_DFD3_06943.t01;Gm_DFD3_07263.t01;Gm_DFD3_07782.t01;Gm_DFD3_08533.t01;Gm_DFD3_10025.t01;Gm_DFD3_10092.t01;Gm_DFD3_09252.t01;Gm_DFD3_12723.t01;Gm_DFD3_13033.t01;Gm_DFD3_13103.t01;Gm_DFD3_13599.t01;Gm_DFD3_14129.t01;Gm_DFD3_15089.t01;Gm_DFD3_10097.t01;Gm_DFD3_10427.t01;Gm_DFD3_10517.t01;Gm_DFD3_11002.t01;Gm_DFD3_11670.t01;Gm_DFD3_16297.t01;Gm_DFD3_16451.t01;Gm_DFD3_16513.t01;Gm_DFD3_16678.t01;Gm_DFD3_17614.t01;Gm_DFD3_17793.t01;Gm_DFD3_18142.t01;Gm_DFD3_18218.t01;Gm_DFD3_18512.t01;Gm_DFD3_19141.t01;Gm_DFD3_15092.t01;Gm_DFD3_19257.t01;Gm_DFD3_19675.t01;Gm_DFD3_22741.t01;Gm_DFD3_23571.t01;Gm_DFD3_24402.t01;Gm_DFD3_25707.t01;Gm_DFD3_25944.t01;Gm_DFD3_26367.t01;Gm_DFD3_26614.t01;Gm_DFD3_26656.t01;Gm_DFD3_26940.t01;Gm_DFD3_27859.t01;Gm_DFD3_27871.t01;Gm_DFD3_28335.t01;Gm_DFD3_31070.t01 ;Gm_DFD3_31127.t01 ;Gm_DFD3_31213.t01 ;Gm_DFD3_31279.t01;Gm_DFD3_31998.t01;Gm_DFD3_32078.t01;Gm_DFD3_32264.t01;Gm_DFD3_32763.t01;Gm_DFD3_33376.t01;Gm_DFD3_33621.t01;Gm_DFD3_33969.t01;Gm_DFD3_34699.t01;Gm_DFD3_35137.t01;Gm_DFD3_35870.t01;Gm_DFD3_36008.t01;Gm_DFD3_36075.t01;Gm_DFD3_36175.t01;Gm_DFD3_36282.t01;Gm_DFD3_36351.t01;Gm_DFD3_37319.t01;Gm_DFD3_37900.t01;Gm_DFD3_38027.t01;Gm_DFD3_38146.t01;Gm_DFD3_39248.t01;Gm_DFD3_39439.t01;Gm_DFD3_39494.t01;Gm_DFD3_39581.t01;Gm_DFD3_39812.t01;Gm_DFD3_40131.t01;Gm_DFD3_40547.t01;Gm_DFD3_41205.t01;Gm_DFD3_42446.t01;Gm_DFD3_43043.t01;Gm_DFD3_43807.t01;Gm_DFD3_44013.t01;Gm_DFD3_44081.t01;Gm_DFD3_44094.t01;Gm_DFD3_44102.t01;Gm_DFD3_44112.t01;Gm_DFD3_44231.t01;Gm_DFD3_44343.t01;Gm_DFD3_45208.t01;Gm_DFD3_45994.t01;Gm_DFD3_46401.t01;Gm_DFD3_47498.t01;Gm_DFD3_47505.t01;Gm_DFD3_47589.t01;Gm_DFD3_20302.t01;Gm_DFD3_21355.t01;Gm_DFD3_03165.t01;Gm_DFD3_20408.t01;Gm_DFD3_22510.t01;Gm_DFD3_22230.t01 |
| DYW | 142 | Gm_DFD3_00109.t01;Gm_DFD3_00113.t01;Gm_DFD3_00451.t01;Gm_DFD3_01977.t01;Gm_DFD3_02012.t01;Gm_DFD3_02030.t01;Gm_DFD3_02040.t01;Gm_DFD3_02777.t01;Gm_DFD3_03046.t01;Gm_DFD3_03191.t01;Gm_DFD3_03437.t01;Gm_DFD3_03578.t01;Gm_DFD3_03680.t01;Gm_DFD3_03866.t01;Gm_DFD3_04010.t01;Gm_DFD3_05507.t01;Gm_DFD3_05756.t01;Gm_DFD3_06459.t01;Gm_DFD3_06614.t01;Gm_DFD3_06832.t01;Gm_DFD3_07193.t01;Gm_DFD3_07292.t01;Gm_DFD3_07737.t01;Gm_DFD3_07934.t01;Gm_DFD3_08293.t01;Gm_DFD3_08712.t01;Gm_DFD3_09462.t01;Gm_DFD3_09628.t01;Gm_DFD3_10450.t01;Gm_DFD3_10569.t01;Gm_DFD3_10758.t01;Gm_DFD3_10779.t01;Gm_DFD3_11519.t01;Gm_DFD3_11559.t01;Gm_DFD3_11560.t01;Gm_DFD3_12129.t01;Gm_DFD3_13106.t01;Gm_DFD3_13490.t01;Gm_DFD3_14329.t01;Gm_DFD3_14427.t01;Gm_DFD3_14726.t01;Gm_DFD3_14767.t01;Gm_DFD3_14997.t01;Gm_DFD3_15594.t01;Gm_DFD3_15867.t01;Gm_DFD3_16169.t01;Gm_DFD3_16184.t01;Gm_DFD3_16367.t01;Gm_DFD3_16618.t01;Gm_DFD3_16653.t01;Gm_DFD3_17694.t01;Gm_DFD3_17755.t01;Gm_DFD3_17986.t01;Gm_DFD3_18046.t01;Gm_DFD3_18394.t01;Gm_DFD3_18901.t01;Gm_DFD3_19240.t01;Gm_DFD3_19561.t01;Gm_DFD3_19562.t01;Gm_DFD3_19591.t01;Gm_DFD3_19600.t01;Gm_DFD3_19654.t01;Gm_DFD3_20242.t01;Gm_DFD3_21933.t01;Gm_DFD3_22743.t01;Gm_DFD3_23416.t01;Gm_DFD3_24396.t01;Gm_DFD3_24933.t01;Gm_DFD3_25031.t01;Gm_DFD3_25454.t01;Gm_DFD3_25461.t01;Gm_DFD3_25476.t01;Gm_DFD3_27399.t01;Gm_DFD3_27790.t01;Gm_DFD3_27912.t02;Gm_DFD3_28762.t01;Gm_DFD3_28873.t01;Gm_DFD3_29074.t01;Gm_DFD3_29400.t01;Gm_DFD3_29405.t01;Gm_DFD3_29901.t01;Gm_DFD3_30385.t01;Gm_DFD3_30913.t01;Gm_DFD3_30935.t01;Gm_DFD3_31510.t01;Gm_DFD3_31892.t01;Gm_DFD3_32951.t01;Gm_DFD3_33074.t01;Gm_DFD3_33385.t01;Gm_DFD3_34933.t01;Gm_DFD3_35304.t01;Gm_DFD3_35467.t01;Gm_DFD3_36114.t01;Gm_DFD3_36763.t01;Gm_DFD3_37310.t01;Gm_DFD3_37508.t01;Gm_DFD3_37652.t01;Gm_DFD3_37664.t01;Gm_DFD3_37939.t01;Gm_DFD3_38153.t01;Gm_DFD3_39076.t01;Gm_DFD3_39186.t01;Gm_DFD3_39428.t01;Gm_DFD3_39553.t01;Gm_DFD3_40268.t01;Gm_DFD3_40526.t01;Gm_DFD3_41133.t01;Gm_DFD3_41443.t01;Gm_DFD3_41519.t01;Gm_DFD3_41925.t01;Gm_DFD3_42687.t01;Gm_DFD3_42753.t01;Gm_DFD3_42930.t01;Gm_DFD3_43910.t01;Gm_DFD3_44212.t01;Gm_DFD3_44337.t01;Gm_DFD3_44704.t01;Gm_DFD3_45298.t01;Gm_DFD3_45641.t01;Gm_DFD3_46232.t01;Gm_DFD3_46911.t01;Gm_DFD3_47653.t01;Gm_DFD3_47845.t01;Gm_DFD3_48061.t01;Gm_DFD3_22402.t01;Gm_DFD3_20655.t01;Gm_DFD3_22605.t01;Gm_DFD3_22918.t01;Gm_DFD3_22181.t01;Gm_DFD3_22274.t01;Gm_DFD3_22023.t01;Gm_DFD3_22269.t01;Gm_DFD3_00033.t01;Gm_DFD3_03332.t01;Gm_DFD3_04099.t01;Gm_DFD3_10735.t01;Gm_DFD3_15605.t01;Gm_DFD3_16326.t01;Gm_DFD3_17944.t01;Gm_DFD3_24765.t01;Gm_DFD3_35017.t01;Gm_DFD3_48132.t01 |
| E | 47 | Gm_DFD3_01767.t01;Gm_DFD3_06831.t01;Gm_DFD3_08294.t01;Gm_DFD3_08308.t01;Gm_DFD3_08446.t01;Gm_DFD3_08736.t01;Gm_DFD3_08888.t01;Gm_DFD3_09361.t01;Gm_DFD3_10929.t01;Gm_DFD3_11974.t01;Gm_DFD3_12336.t01;Gm_DFD3_12613.t01;Gm_DFD3_13263.t01;Gm_DFD3_13413.t01;Gm_DFD3_13527.t01;Gm_DFD3_15975.t01;Gm_DFD3_16955.t01;Gm_DFD3_17217.t01;Gm_DFD3_18209.t01;Gm_DFD3_20076.t01;Gm_DFD3_21005.t01;Gm_DFD3_21018.t01;Gm_DFD3_22862.t01; Gm_DFD3_24001.t01;Gm_DFD3_24858.t01;Gm_DFD3_25050.t01;Gm_DFD3_26041.t01;Gm_DFD3_26146.t01;Gm_DFD3_26524.t01;Gm_DFD3_27828.t01;Gm_DFD3_29452.t01;Gm_DFD3_29948.t01;Gm_DFD3_31420.t01;Gm_DFD3_32758.t01;Gm_DFD3_33515.t01;Gm_DFD3_37062.t01;Gm_DFD3_37077.t01;Gm_DFD3_38702.t01;Gm_DFD3_39473.t01;Gm_DFD3_40692.t01;Gm_DFD3_43148.t01;Gm_DFD3_43516.t01;Gm_DFD3_44712.t01;Gm_DFD3_46291.t01;Gm_DFD3_21571.t01;Gm_DFD3_21746.t01;Gm_DFD3_22709.t01 |

**Supplementary Table 5 Screen quantitative gene information**

|  | log₂FC | TPM | Positioning |
| --- | --- | --- | --- |
| *Gm_DFD3_04099* | 3.3 | 14.56 | chlo |
| *Gm_DFD3_00451* | -3.1 | 23.45 | chlo |
| *Gm_DFD3_31510* | 2.4 | 30.14 | plas |
| *Gm_DFD3_46232* | 1.9 | 11.7 | chlo |
| *Gm_DFD3_48132* | -1.8 | 1.2 | chlo |

- 1. **Supplementary Figure**

**
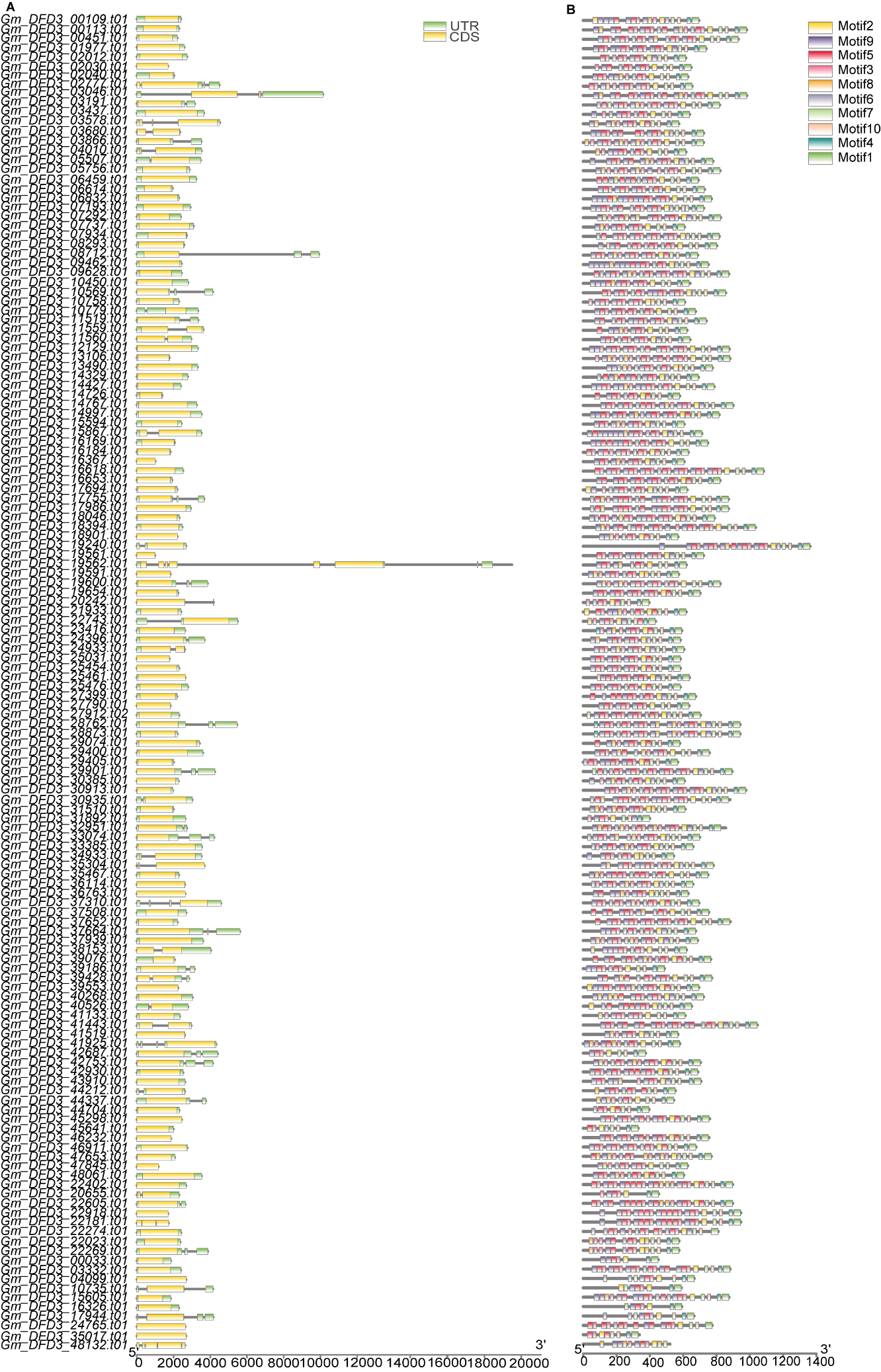
**

**Supplementary Figure 1.** Soybean DYW-type PPR gene sequence structure and motif analysis. (**A**) Schematic diagram of the gene structure of soybean DYW-type PPR gene sequences. (**B**) Schematic diagram of conserved domain analysis of soybean DYW-type PPR gene sequences.


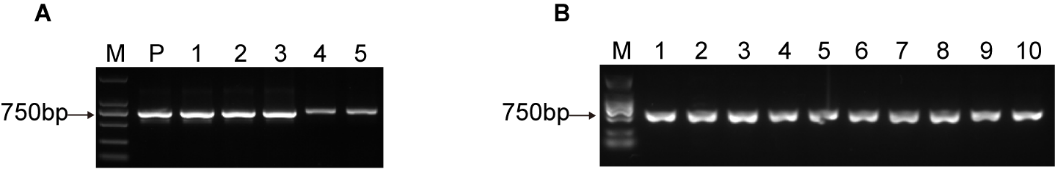


**Supplementary Figure 2.** Knockout Positive Roots and *ndhD*-878 Editing Efficiency PCR Verification. (**A**) PCR verification of knockout positive roots, M: DNA marker 2K Plus II; P: PCR verification of empty vector plant roots; 1-5: PCR verification of knockout positive roots. (**B**) PCR gel image verifying the *ndhD*-878 RNA editing site, M: DNA marker 2K Plus II; 1-2: empty vector samples; 3-4: empty vector drought samples; 5-6: knockout samples; 7-10: knockout drought samples.
